# DFT Investigations and Molecular Docking as Potent Inhibitors of SARS-CoV-2 Main Protease of Novel Pyrimidine Dione Derivatives

**DOI:** 10.1155/bri/7961294

**Published:** 2025-08-11

**Authors:** Wesam S. Shehab, Jalal Hasan Mohammed, Naja Magdy, Wael A. Zordok, Mariusz Jaremko, Doaa A. Elsayed

**Affiliations:** ^1^Department of Chemistry, Faculty of Science, Zagazig University, Zagazig 44519, Egypt; ^2^Department of Pharmaceutical Chemistry, Faculty of Pharmacy, Al-Farahidi University, Baghdad, Iraq; ^3^Department of Pharmaceutical Chemistry, Faculty of Pharmacy, University of Karbala, Karbala, Iraq; ^4^Biological and Environmental Science and Engineering (BESE), King Abdullah University of Science and Technology (KAUST), Thuwal 23955-6900, Saudi Arabia

**Keywords:** ADME properties, COVID-19, diphenyl derivative, imidazolopyrimidine, pyranopyrimidine, pyridotriazine

## Abstract

The global outbreak of SARS-CoV-2 has emerged as a major public health crisis due to its rapid transmission and significant morbidity and mortality rates. In response, there is an urgent need to discover novel antiviral agents targeting key viral proteins. In this study, a new series of pyrimidine-2,4-dione derivatives was synthesized from barbituric acid and its thio analog, aiming to explore their potential inhibitory activity against the SARS-CoV-2 main protease (Mpro). The synthesis involved 1,4-addition and cyclodehydration reactions, yielding novel pyran and condensed pyrimidine derivatives. The chemical structures were confirmed using various spectroscopic techniques. Molecular docking studies were performed using MOE software (version 2022) targeting Mpro (PDB ID: 6LU7), revealing favorable binding affinities for several compounds. Compound 9 showed the best docking score (−12.70 kcal/mol), followed by Compound 4 (−12.38 kcal/mol) and Compound 11 (−12.13 kcal/mol), with key interactions involving residues such as Asn142, His41, and Glu166. DFT calculations were carried out to evaluate electronic properties, including energy gap (ΔE), global hardness (η), and softness (σ), indicating that Compound 10 was the most reactive with notable HOMO–LUMO characteristics. Additionally, the synthesized compounds were screened for drug-likeness based on Lipinski's rule of five and ADME parameters. Overall, the study identifies promising pyrimidine-based inhibitors of SARS-CoV-2 Mpro and provides valuable insights for further optimization and development of potential antiviral agents.

## 1. Introduction

Since the emergence of SARS-CoV-2 in late 2019, the world has faced an unprecedented public health challenge marked by high morbidity and mortality rates. The virus, classified as a positive-sense single-stranded RNA virus of the Coronaviridae family, encodes several structural and nonstructural proteins essential for viral replication and pathogenesis. Among these, the main protease (Mpro or 3CLpro) and RNA-dependent RNA polymerase (RdRp) have emerged as validated drug targets due to their indispensable role in processing viral polyproteins and RNA synthesis, respectively [1–5]. Despite significant global efforts, the current therapeutic arsenal remains limited, and the emergence of new variants underscores the urgent need to develop novel antiviral agents. Structure-based drug design (SBDD) and in silico screening have become indispensable tools for identifying promising lead compounds targeting SARS-CoV-2 proteins [6, 7]. Several chemical scaffolds have been explored for their potential antiviral properties, including nucleoside analogs [8], thiazolidinones [9], pyrimidines [10], oxazolone derivatives [11], and fused heterocyclic compounds [12]. Oxazolone-based compounds, known for their versatile synthetic reactivity and wide-ranging pharmacological applications, have gained increasing attention in antiviral drug discovery. Oxazolone rings serve as intermediates in synthesizing numerous biologically active molecules, including antimicrobial, anticancer, antidiabetic, and antiviral agents [12, 13]. Their electron-deficient nature and multiple sites for nucleophilic attack make them excellent candidates for designing hybrid heterocycles with improved bioactivity [14]. In recent years, pyrimidine-2,4-diones and their thio analogs have also attracted considerable interest due to their structural similarity to natural nucleobases and their ability to inhibit viral enzymes by mimicking substrate binding. Notably, compounds bearing pyrimidine and fused pyran rings have demonstrated inhibitory effects on SARS-CoV-2 Mpro in several molecular docking and simulation studies [15–17]. Moreover, molecular dynamic (MD) simulations and density functional theory (DFT) calculations have provided valuable insights into the binding stability and electronic reactivity of these inhibitors [18–20].

Several recent studies have highlighted the antiviral potential of diverse small molecules against SARS-CoV-2 using computational and experimental approaches. For instance, Sutradhar et al. reported the synthesis and docking of lauroyl thymidine analogs as Mpro inhibitors, which showed stable interactions with key catalytic residues [21]. Similarly, thiazolidine-4-one derivatives exhibited promising binding affinities toward the Mpro active site and showed good ADMET properties [22]. Cytidine-based compounds targeting RdRp have also demonstrated significant antiviral potential, supported by pharmacokinetic predictions and POM analyses [23]. Furthermore, molecules synthesized based on oxazolone frameworks have shown selective inhibition of viral targets, including SARS-CoV-2 Mpro, as demonstrated in the work by Mariappan et al. [11], where docking and dynamics studies revealed key hydrogen bonding and hydrophobic interactions within the enzyme's binding cleft. Likewise, recent reports by Elsayed et al. and Shehab et al. incorporated computational modeling and DFT analyses to design quinoline and pyrimidine derivatives with dual inhibitory activities against viral enzymes and acceptable pharmacokinetic profiles [24–28]. Given the structural versatility and documented bioactivities of oxazolones and pyrimidines, we aimed in the present study to design and synthesize a novel series of heterocyclic derivatives via cyclocondensation reactions involving oxazolone scaffolds, barbituric acid, and their thio analogs. The chemical structures of the newly synthesized compounds were confirmed by spectroscopic methods (IR and NMR), and their in silico binding potentials were assessed through molecular docking studies against the SARS-CoV-2 Mpro (PDB ID: 6LU7). DFT calculations were conducted to evaluate the frontier molecular orbitals, electronic properties, and reactivity descriptors, providing a theoretical basis for compound activity. Additionally, drug-likeness and pharmacokinetic properties were predicted using Lipinski's rule and ADME profiling. Collectively, this study presents a comprehensive strategy combining green synthetic chemistry, computational modeling, and bioinformatics tools to identify novel SARS-CoV-2 inhibitors.

## 2. Results and Discussion

### 2.1. Chemistry

**Scheme 1:**
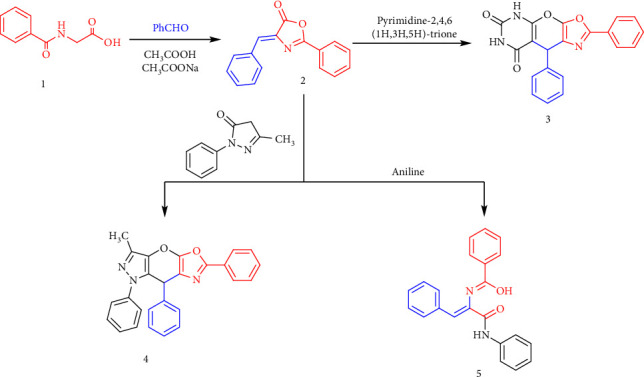
Design of heterocyclic systems from benzylidene oxazolone derivative.

Our recent research focused on the synthesis and chemical behavior of the benzylidene oxazolone derivative 2, designed to act as a Michael acceptor or acylating agent. When treated with carbon nucleophiles from various heterocyclic systems, 4-benzylidene-2-phenyloxazol-5(4H)-one (2) undergoes a Michael addition reaction to form an adduct, which may either remain acyclic or undergo intramolecular cyclization. The reaction of barbituric acid with Compound 2 led to the formation of a conjugate addition product identified as 2,9-diphenyl-5,9-dihydro-6H-oxazolo[4′,5′:5,6]pyrano[2,3-d]pyrimidine-6,8(7H)-dione (3). The ^1^H NMR spectrum of Compound 3 exhibited a broad downfield signal at *δ* = 13.12 ppm, corresponding to the two NH protons. Additionally, its FT-IR spectrum showed a characteristic absorption band at 3271 cm^−1^, confirming the presence of NH and C=O groups (Scheme 1).

**Scheme 2:**
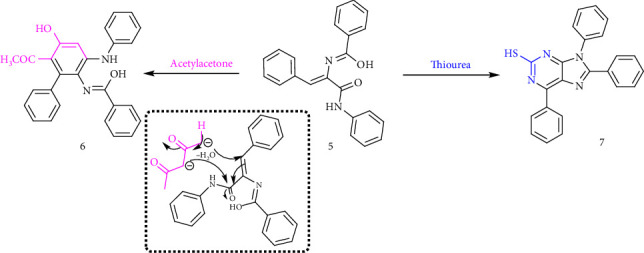
The cyclization reaction of N-phenylpyrazolone with oxazolone derivatives.

The cyclization reaction of N-phenyl pyrazolone with oxazolone derivative (2) afforded the pyrazolo-oxazolopyran compound, identified as 3-methyl-1,6,8-triphenyl-1,8-dihydropyrazolo[3′,4′:5,6]pyrano[3,2-d]oxazole (4). The IR spectrum of Compound 4 confirmed successful cyclization through the presence of characteristic absorption bands attributed to the pyran moiety. In contrast, the reaction of oxazolone (2) with aniline yielded the N-phenyl cinnamamide derivative (5). The ^1^H NMR spectrum of Compound 5 exhibited two deshielded singlets at *δ* = 10.09 ppm and *δ* = 9.22 ppm, corresponding to the OH and NH protons, respectively. The IR spectrum also showed a strong band corresponding to the C=O stretching vibration. Subsequent addition of acetylacetone to Compound 5, followed by cyclodehydration, led to ring closure and oxidation, affording Compound 6. The ^1^H NMR spectrum of 6 displayed three downfield singlets at *δ* = 9.22, 10.05, and 11.97 ppm, corresponding to the NH, OH, and phenolic OH protons, respectively. Moreover, the reaction of the *α*,β-unsaturated system (5) with thiourea via cyclocondensation furnished the pyrimidine derivative (7). The structure of 7 was confirmed by the appearance of a downfield signal at *δ* = 13.35 ppm in the ^1^H NMR spectrum, assigned to the SH proton, and a characteristic IR absorption at 1216 cm^−1^, indicating the presence of a C=S group (Scheme 2).

**Scheme 3:**
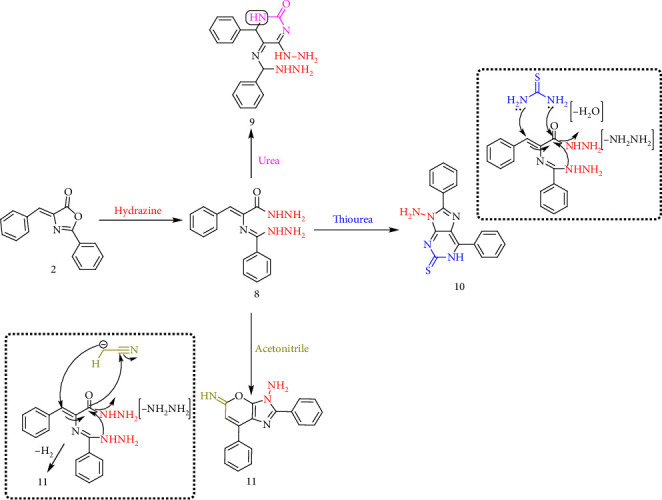
Synthesis of pyrimidine derivatives.

The reaction of Compound **2** with hydrazine hydrate (99%) in absolute ethanol resulted in hydrazinolysis, yielding hydrazide derivative **(8)**. The structure of Compound **8** was confirmed by the presence of four downfield signals in its ^1^H NMR spectrum, corresponding to NH protons, along with a C=O stretching frequency in its IR spectrum. Subsequent cyclization of hydrazide **8** with urea afforded pyrimidine derivative **(9)**. The structure of Compound **9** was verified by ^1^H NMR spectroscopy, which exhibited four deshielded signals attributed to NH protons, while the IR spectrum displayed a characteristic C=O stretching frequency. As illustrated in Scheme 3, thiourea underwent 1,4 addition to the α,β-unsaturated system, followed by elimination of aniline and oxidation, producing imidazopyrimidine derivative **(10)**. The ^1^H NMR spectrum of Compound **10** showed two downfield signals assigned to NH protons, while the ^13^CNMR spectrum exhibited a carbon signal at 180.66 ppm for the C=S carbon. Additionally, the IR spectrum confirmed the presence of the C=S group with a stretching frequency at 1240 cm^−1^. The detailed analysis is provided in Supporting Figures 1–31.

### 2.2. In Silico Studies

In silico studies and related methods are crucial for assessing various physicochemical properties and predicting a molecule's biological activity, ADME parameters, and toxicity. Compounds **3**, **4**, **9**, **10**, and **11** were evaluated for modified ADME characteristics using the SwissADME and pkCSM ADMET web server tools, and their interactions with the COVID-19 main protease were analyzed through molecular docking with the N3 inhibitor complex (PDB ID: 6LU7).

### 2.3. Analysis of Ligand Characteristics

In this study, the drug-likeness of the synthesized compounds was assessed using Lipinski's rule of five (RO5), a well-established guideline that predicts the oral bioavailability of compounds based on key physicochemical properties such as molecular weight (≤ 500 Da), partition coefficient logP (≤ 5), number of hydrogen bond donors (≤ 5), and hydrogen bond acceptors (≤ 10). As summarized in Table 1, all five selected ligands (Compounds 3, 4, 9, 10, and 11) fully complied with RO5 criteria, exhibiting no violations. In contrast, the native ligand N3 co-crystallized with Mpro (PDB ID: 6LU7) displayed two violations, with a molecular weight of 680 Da and 12 hydrogen bond acceptors. These results are consistent with previous computational and experimental studies that emphasize the significance of RO5 compliance in the early stages of antiviral drug development. For example, Vijayakumar et al. and Silva Arouche et al. have demonstrated that molecules adhering to RO5 tend to have superior pharmacokinetic and absorption profiles, which are crucial for effective SARS-CoV-2 inhibition [2, 23]. Similarly, a study by Khaerunnisa reported that natural products with favorable RO5 profiles exhibited stronger binding affinities and better drug-likeness scores when docked into the active site of SARS-CoV-2 Mpro [3, 23] (Table 1). Thus, in light of these literature reports, our findings suggest that the selected compounds are not only structurally suitable for drug development but may also possess favorable ADME properties. The absence of any RO5 violations in these ligands highlights their potential as orally active antiviral agents. In particular, Compound 10 (C_17_H_13_N_5_S) showed the lowest molecular weight and ideal hydrogen bonding profile, which supports its potential as a lead compound in future optimization studies.

### 2.4. Analysis of ADME Characteristics of Ligand

Table 2 shows the findings of our subsequent inquiry into the ADME pharmacokinetics, absorption, distribution, excretion, and toxicity of several bioactive chemicals using the SwissADME and admetSAR service [29]. Except for N3, 3, 9, and 10, all compounds exhibited polar surface areas below 100, suggesting potentially enhanced membrane permeability or improved oral absorption. Among the selected compounds, N3 exhibited lower intestinal absorption compared to compounds 3 and 4, which showed higher intestinal absorption rates of 94.7% and 98.8%, respectively. Skin permeability is assessed by the Log Kp value; a compound with a Log Kp value greater than 2.5 is considered to have low skin permeability. All the compounds were predicted to have low skin permeability, as their Log Kp values exceeded 2.5. The permeability of a compound across the blood–brain barrier (BBB) is evaluated using the LogBB value. A LogBB value greater than 0.3 indicates strong BBB permeability, values between 0.3 and 1 suggest moderate BBB permeability, and a LogBB value of less than 1 indicates relatively poor BBB permeability [30].

### 2.5. Ramachandran Plot

**Figure 1:**
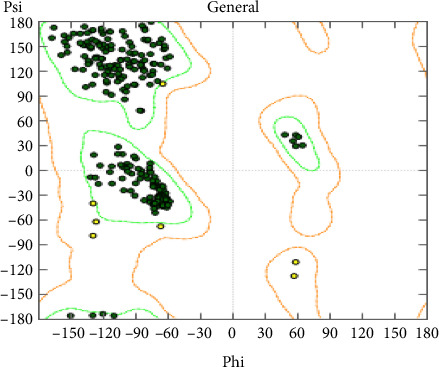
Ramachandran plot.

To validate the stereochemical quality of the modeled protein structure, a Ramachandran plot was generated using MOE. The plot shows the distribution of the phi (φ) and psi (ψ) dihedral angles of the protein backbone. As shown in Figure 1, the majority of the residues fall within the most favored (green) and allowed (orange) regions, indicating good stereochemical quality of the model. Only a few residues were found in disallowed regions, which suggests that the structure is overall stable and suitable for molecular docking and further computational studies.

### 2.6. Molecular Docking

Molecular docking analysis was conducted to examine the interaction of natural compounds with various anti-COVID-19 molecular targets. This analysis was used to explore the potential mechanisms of action for newly developed anti-COVID-19 drugs, comparing them to the standard reference N3 inhibitor, N-[(5-methylisoxazol-3-yl)carbonyl]-(1R,2Z)-4-(benzyloxy)[(3R)-2- oxopyrrolidin-3-yl]-4-oxo-1-(methylbutenyl)-L-leucinamide (PDB code: 6LU7).

The selection of SARS-CoV-2 main protease (Mpro, PDB ID: 6LU7) was based on its crucial role in mediating viral replication and transcription by cleaving polyproteins translated from the viral RNA. As an essential enzyme in the life cycle of SARS-CoV-2, Mpro has emerged as a well-validated drug target for the development of COVID-19 therapeutics. Furthermore, the availability of its high-resolution crystal structure complexed with the N3 inhibitor allows accurate modeling of the active site, making it highly suitable for SBDD approaches, including molecular docking.

**Figure 2:**
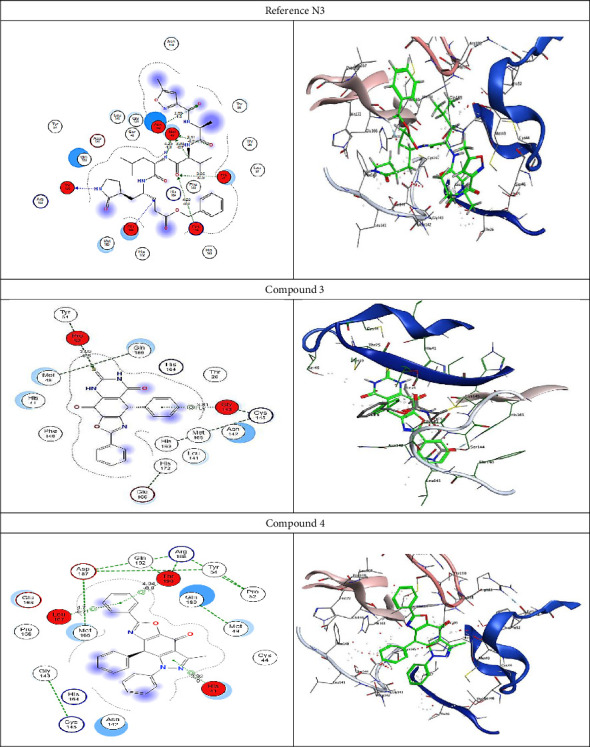
2D and 3D receptor interactions between the potential produced drugs and COVID-19 targets.

**Figure 3:**
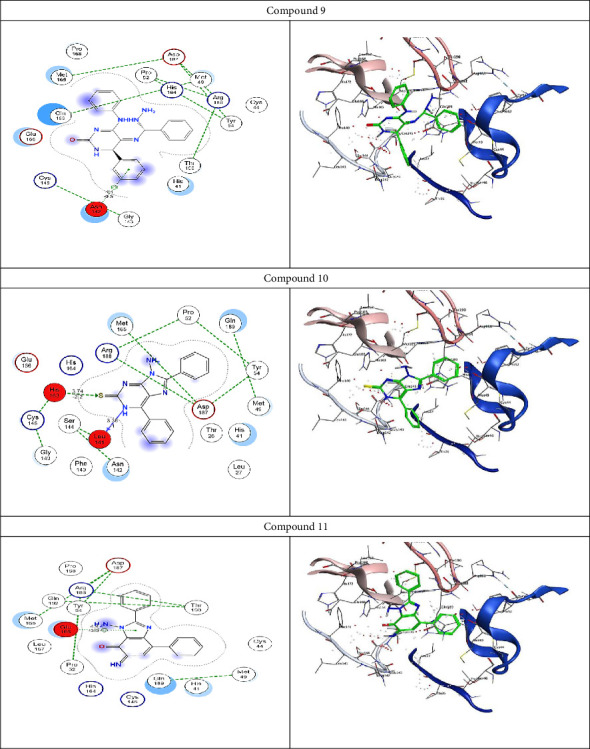
2D and 3D receptor interactions of prospective anti-COVID-19 drugs.

To validate the docking results at the active site, the co-crystallized ligand N3 was re-docked using the same set of parameters. The root mean square deviation (RMSD) of the best-docked pose was 1.8721 Å, with an energy score of −15.2100 kcal/mol, corroborating the MOE software's docking results. As shown in Figure 2, N3 formed 8 hydrogen bonds with Met49, Thr190, Asn142, His41, Cys145, and Glu166.  Table 3 shows that Compound 3 bound to the active site with a docking score of −11.5558 kcal/mol. Compound 4 formed two pi-H-bonds with Thr190 and His41, while Compounds 9, 10, and 11 achieved the highest docking scores of −12.7032 and −10.5335 kcal/mol, respectively. Their re-docking poses are depicted in Figures 2 and 3.

### 2.7. The Structure–Activity Relationship (SAR)

The SAR analysis of the synthesized pyrimidine derivatives clearly indicates that the nature, position, and spatial arrangement of substituents significantly impact their binding affinity. Compound 9, characterized by three phenyl rings attached to a nonplanar pyrimidine core, demonstrated a very strong docking score (−12.70 kcal/mol). This can be attributed primarily to the extensive aromatic surface area which enhances π–π stacking interactions within the active site, as also reported in similar pyrimidine derivatives targeting kinase enzymes [31, 32]. However, the nonplanarity of the molecule likely induces steric hindrance and spatial crowding that limit optimal hydrogen bonding with key residues such as Asn142, resulting only in π–H interactions. This highlights the delicate balance between aromaticity and molecular geometry in drug–target interactions [33, 34]. In comparison, Compound 10 adopts a more planar geometry with enhanced electronic polarization due to the presence of polar NH–NH_2_ groups. This planar structure is generally favorable for binding as it facilitates proper orientation and closer contact with the binding pocket [34]. However, despite this, Compound 10 exhibited a reduced docking score (−10.53 kcal/mol) relative to 9, which can be explained by the limited number of strong hydrogen bonds formed with residues such as Leu141 and His163. This suggests that merely increasing planarity and polarity does not guarantee improved binding; the spatial positioning of substituents must also allow effective interaction with key amino acids [35]. Compound 11 showed a high docking affinity (−12.13 kcal/mol), which correlates with the presence of a pyrazole ring substituted with a free NH_2_ group acting as a hydrogen bond donor. The well-positioned NH_2_ group enables formation of strong hydrogen bonds and favorable electrostatic interactions with residues like Glu166, which has been emphasized in recent SAR studies on pyrazole-containing inhibitors [36]. This underlines the importance of polar substituents that are capable of specific hydrogen bonding in increasing target affinity and biological efficacy [36]. Further comparison of Compounds 3 and 4 reveals that aromatic substitution patterns also critically influence activity. Compound 4, containing a fused phenyl–pyrazole–pyrimidine system, exhibited better docking energy (−12.38 kcal/mol) than the simpler Compound 3 (−11.55 kcal/mol). This enhancement can be attributed to improved π–π stacking and hydrophobic interactions enabled by extended conjugation and planarity, consistent with observations in multiring fused heterocycles [37]. The positional effects of aromatic substituents allow optimized spatial occupancy within the hydrophobic pockets of the enzyme active site, maximizing van der Waals and electrostatic contacts [38]. Overall, these findings underscore that a combination of aromatic surface area, planarity, hydrogen bond donor/acceptor availability, and steric accessibility governs the binding efficacy of these pyrimidine derivatives. Designing future analogs with strategically positioned polar substituents, preserving planarity while minimizing steric clashes, is likely to yield compounds with superior biological activities. Such SAR insights align well with recent literature emphasizing the crucial role of substituent optimization in small-molecule inhibitors [39].

### 2.8. Computational Details

#### 2.8.1. Computational Method

Geometric parameters and energies were calculated using DFT, a quantum mechanical modeling method used to investigate the electronic structure of molecules and condensed matter systems. The calculations were performed at the B3LYP/CEP-31G level of theory, implemented in the GAUSSIAN 98W software package [40]. Geometries were optimized with the CEP-31G basis set to achieve high accuracy in energy calculations. Atomic charges were derived from natural atomic orbital populations. The B3LYP functional is a hybrid approach [41] that combines the gradient-corrected functional proposed by Becke [42] and Lee et al. [43], with the Hartree–Fock local exchange function [44].

#### 2.8.2. Structural Parameters and Models

##### 2.8.2.1. Compound (3)

###### 2.8.2.1.1. 2,9-Diphenyl-6-thioxo-5,6,7,9-tetrahydrooxazolo[4,5-g]quinazoline-4,8-dione

**Figure 4:**
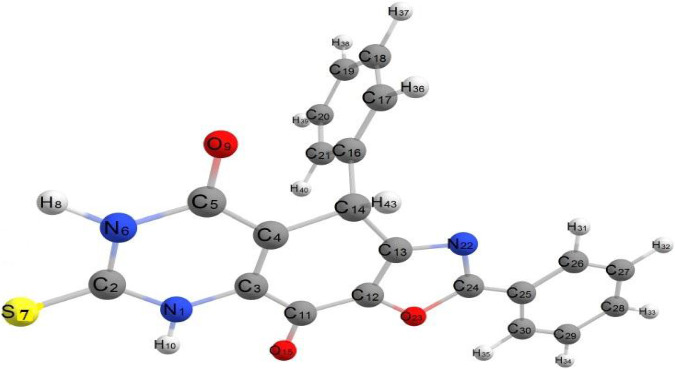
Optimized geometrical structure of Compound 3 by using DFT calculations.

The molecule in question exhibits relatively low steric hindrance and consists of two aromatic systems: two phenyl rings and a fused system described as 1,6-thioxo-5,6,7,9-tetrahydrooxazolo[4,5-g]quinazoline-4,8-dione. As observed in the optimized structure of molecule (3), the fused system along with one phenyl ring lies in the same plane, while the other phenyl ring is oriented perpendicular to this plane. The molecule exhibits nonplanarity, with the two planes being perpendicular to each other. The dihedral angles C13–C14–C4–C5 and N1–C3–C11–C12 are 175.51° and −176.88°, respectively, whereas the dihedral angles C24–N22–C13–C14 and C11–C12–O23–C24 are 176.30° and −178.51°, respectively. These dihedral angles confirm that the fused aromatic ring system is entirely planar. Additionally, the dihedral angles N22–C24–C25–C26, O23–C24–C25–C30, and C30–C25–C24–N22 are −0.205°, 0.150°, and 179.62°, respectively. These near-zero or near-180° values suggest that the fused aromatic ring and the phenyl ring are aligned in the same plane. Conversely, the dihedral angles C13–C14–C16–C21 and C13–C14–C16–C17 are 62.79° and −116.92°, respectively, indicating that the phenyl group is positioned out of the plane occupied by the rest of the molecule, as illustrated in Figure 4. The bond angles around carbon, nitrogen, and oxygen atoms in the molecule range from 108.15° to 125.79°, reflecting predominant sp2 hybridization across most of the molecule, except for nitrogen atoms which exhibit sp3 hybridization. All bond angles and dihedral angles are listed in Table 1S. The molecule's nonplanarity affects its biological activity; the phenyl ring, perpendicular to the plane, can rotate around the C14–C16 bond of the fused aromatic system. The compound's energy is −147855.296 kcal/mol, and the presence of electron-withdrawing and electron-donating groups results in a weak dipole moment of 3.028 D.

The C-C bond lengths in Compound 3 range from 1.331 to 1.516 Å, representing the shortest C-C bonds in the molecule [45]. The C-N bond lengths range from 1.335 to 1.372 Å [46] which are notably longer than the C-O bond lengths, which vary from 1.208 to 1.366 Å [44]. These bonds exhibit single-bond character for nitrogen and oxygen [47]. The C2–S7 bond length is 1.692 Å [48], indicative of double-bond character [49]. All bond lengths are detailed in Table 1S and compared with similar structures from crystal data [45]. Further analysis of bond lengths in related heterocyclic compounds can be found elsewhere [49].

Table 1S (supporting information): DFT calculations are used to determine the equilibrium geometric properties of the compound, including bond lengths (Å), bond angles (°), dihedral angles (°), total energy (k cal/mol), and dipole moment (3).

##### 2.8.2.2. Compound 4

###### 2.8.2.2.1. 3-Methyl-1,6,8-triphenyl-1,8-dihydropyrazolo[3′,4′:5,6]pyrano[3,2-d]oxazole

**Figure 5:**
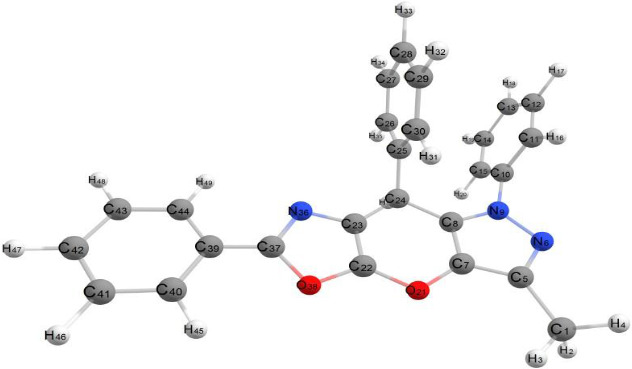
Optimized geometrical structure of Compound 4 by using DFT calculations.

Figure 5 describes the optimal geometrical structures of Compound 4. This molecule is nonplanar, with two separate planes: One contains the 3-methyl-1-phenyl-1,8-dihydropyrazolo[3′,4′:5,6]pyrano[3,2-d]oxazole aromatic system, while the other has the two phenyl rings at positions 6 and 8. The bond lengths in this compound are like those in Compound 3.

The dihedral angles of this substance are as follows: C44–C39–C37–N36 and C44–C39–C37–O38 are roughly 0.00° and 180.00°, respectively, whereas N9–C8–C24–C23 and C39–C37–O38–C22 are −175.97° and −177.73°, respectively. These numbers imply that the phenyl group is located within the plane of the fused aromatic rings. The dihedral angles of C30–C25–C24–C23 and C30–C25–C24–C8 are 63.07° and −55.54°, respectively, whereas C11–C10–N9–C8 and C15–C10–N9–C8 are 99.16° and −78.37°, respectively. These angles corroborate that the two phenyl rings are oriented perpendicularly to the plane of the fused aromatic rings and parallel to one another, as illustrated in Figure 5. The bond angles around the compound's carbon atoms range from 110.01° to 128.79°, while those around the nitrogen atoms range from 122.12° to 126.52°. These results indicate sp^2^ hybridization over most atoms in the molecule. The compound's energy is −155327.314 kcal/mol, which is less than that of Compound 3. Its dipole moment is 1.904 D, which is less than that of Compound 3 due to the lack of polarity and the compound's geometric structure.  Table 2S lists the bond lengths for C-C, C-N, and C-O bonds, which are comparable to those discovered in Compound 4.

Table 2S (supporting information): DFT calculations are used to determine the equilibrium geometric properties of the compound, including bond lengths (Å), bond angles (°), dihedral angles (°), total energy (k cal/mol), and dipole moment (4).

##### 2.8.2.3. Compound 9

###### 2.8.2.3.1. 6-Phenyl-4-(phenylamino)-5-(2-phenyltriazaneylidene)-5,6-dihydropyrimidin-2(1H)-one

**Figure 6:**
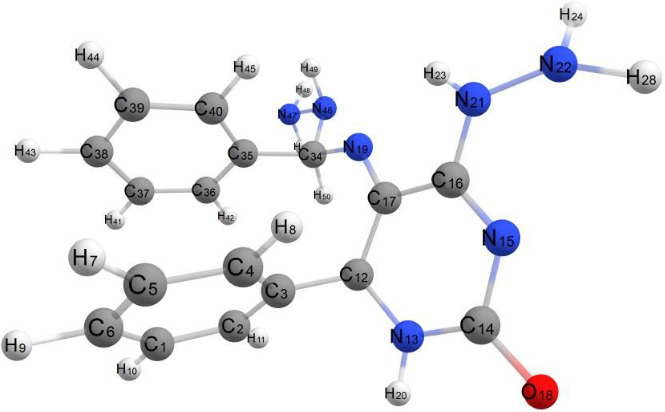
Optimized geometrical structure of Compound 9 by using DFT calculations.

Figure 6 shows the optimized geometrical structure of Compound **9**. Unlike the previously studied Compounds **3** and **4**, Compound **9** lacks a fused aromatic system. Instead, it consists of three phenyl rings attached to a pyrimidine ring. These structural features render Compound **9** completely nonplanar. Consequently, the molecule is expected to exhibit a higher dipole moment compared to the other compounds.

The dihedral angles N15–C16–N21–C22 (20.48°), C4–C3–C12–C17 (−40.17°), and N19–C34–C35–C40 (38.69°) confirm the molecule's nonplanar geometry, with its fragments distributed across three different planes. The calculated energy of Compound **9** is −153791.243 kcal/mol, which is lower than Compound **3** but higher than Compound **4**. The combination of terminal polar groups and the molecule's nonplanarity results in a dipole moment of 7.966 D, larger than those of the previous compounds, as summarized in Table 3S.

Table 3S (supporting information): DFT calculations are used to determine the equilibrium geometric properties of the compound, including bond lengths (Å), bond angles (°), dihedral angles (°), total energy (k cal/mol), and dipole moment (9).

##### 2.8.2.4. Compound 10

###### 2.8.2.4.1. 9-Amino-6, 8-diphenyl-1,9-dihydro-2H-purine-2-thione

**Figure 7:**
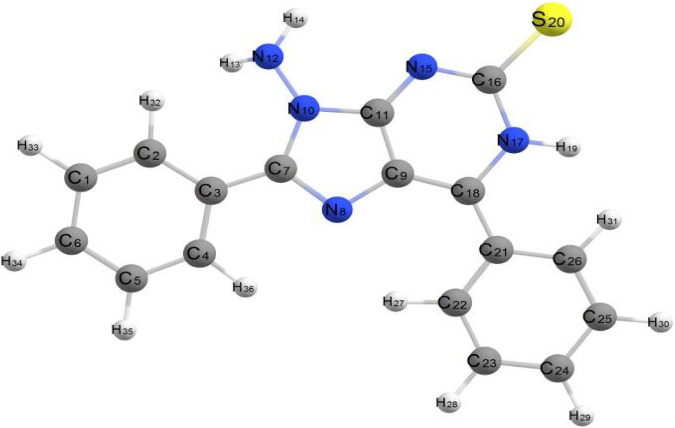
Optimized geometrical structure of Compound 10 by using DFT calculations.

Figure 7 shows the optimized geometry of Compound 10. This chemical differs structurally from the previous ones. The first difference is that this compound is completely planar; all fragments are lying in the same plane and all dihedral angles are varied between two values 0.00° and 180.00° as listed in Table 4S.

Table 4S (supporting information): DFT calculations are used to determine the equilibrium geometric properties of the compound, including bond lengths (Å), bond angles (°), dihedral angles (°), total energy (k cal/mol), and dipole moment (10).

The second difference is the consistent of the compound which composed of fused aromatic system and two phenyl rings attached to the purine fused system in addition to terminal sulfur atom; from the optimized geometrical structure, we can observed that sulfur atom with high polarity lying in opposite direction respect to the bonded two phenyl rings, the planarity of this compound, and the unique feature structure may cause generating of greater dipole. The total energy value of this compound is −114732.671 k cal/mol which is considered as higher value more than the previous studied compounds and considered as less table than others, and also, the dipole moment of this compound is greater than all studied compounds as expected, and the dipole moment is 12.312D.

##### 2.8.2.5. Compound 11

###### 2.8.2.5.1. 5-Imino-2,7-diphenylpyrano[2,3-d]imidazol-3(5H)-amine

**Figure 8:**
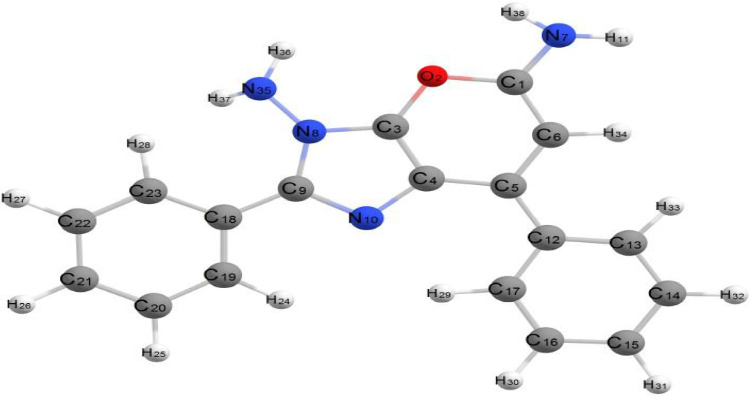
Optimized geometrical structure of Compound 11 by using DFT calculations.

Figure 8 illustrates the optimized geometric structure of Compound 20. This chemical is structurally identical to Compound 17, but with a -NH2 group substituting the sulfur atom linked to the fused aromatic system. The presence of –S atom as C=S causes withdrawing electrons and decreases electron density on the fused aromatic ring system with causing deactivation, while the replacement of C=S by –NH2 which behaves as donating group causes increasing electron density on the fused aromatic ring system and causing activation of the compound.

The presence of donating group reduces the value of dipole moment 3.190 D, and the overall energy value −116566.493 k cal/mol is less than the energy of Compound 11 by 2000 kcal/mol, so this compound is more stable than Compound 11. All structure parameters of this compound are listed in Table 5S.

Table 5S (supporting information): Equilibrium geometric parameters such as bond lengths (Å), bond angles (°), dihedral angles (°), total energy (kcal/mol), and the dipole moment of Compound 11 were obtained using DFT calculations.

### 2.9. Charge Distribution Analysis

The charge distribution analysis for the optimal geometric configurations of all examined compounds was performed using natural population analysis (NPA). This analysis evaluated the charge distribution across the heteroatom's nitrogen, oxygen, and sulfur in the compounds, which serves as an indicator of their polarity.

The chemical reactivity of a molecule is often interpreted in terms of its charge distribution. Although the chemist has an intuitive feeling for the qualitative nature of these charge distributions, assigning atomic charges quantitatively is far from trivial.

Charge distributions are accessible through quantum chemical calculations. However, different methods are available and results seem to be heavily dependent on the quantum mechanical method chosen. Moreover, the charge on an atom in a molecule is not a directly observable experimental quantity, so that a direct comparison of the calculated atomic charges to experiment is impossible. In addition, computing times increase superlinearly (typically quadratically to quartically) with the number of electrons in the molecular system of interest, thus precluding very accurate calculations. Moreover, the practicing chemist likes to hold on to intuitive and easy-to-use chemical concepts in order to interpret the charge distribution.

One of these concepts is electronegativity. Being by far one of the oldest chemical concepts, electronegativity gives a measure for the unequal sharing of electrons in a chemical bond, resulting in bond polarity. Introduced in 1932 by Harris [50] as “the power of an atom in a molecule to attract electrons to itself,” the concept has undergone great changes since then. Many scales were introduced, the most important due to Balbás et al. [51], Gordy [52], Allred and Rochow [53], and Huheey et al. [54]. Despite all the proposed electronegativity scales, a rigorous quantum mechanical definition of the electronegativity concept was clearly lacking. This changed in 1978 when Parr et al. [55], after preliminary work of Iczkowski and Margrave [56], identified the electronegativity with the negative of the Lagrange multiplier from density functional theory, which appears when the energy functional of the electron density is minimized with the constraint of constant number of electrons N.

Compound 11 exhibits a net negative pole due to the presence of polar atoms and its ideal planarity, resulting in a larger dipole moment compared to the other compounds tested. In contrast, the other compounds display lower dipole moments, likely due to the absence of strong positive and negative poles and their nonplanar structures. In Compound 11, there is a significant buildup of charge density on the donating nitrogen, oxygen, and sulfur atoms of 9-amino-6,8-diphenyl-1,9-dihydro-2H-purine-2-thione. The atoms with the highest charges are S20 (−0.726) in the –C=S group, N17 (−0.183), and N15 (−0.304). Additionally, N8 and N10 in the purine ring have charges of −0.324 and −0.012, respectively, while N12 in the terminal amino group has a charge of −0.283, as detailed in Table 4. The variation on the dipole moment value changed from 1.904 D in Compound 4 to 12.312 D in Compound 11; this variation depends on the changing in the charges accumulated on all atoms in the studied compounds, especially heteroatoms; and the strong withdrawing groups cause generating of a negative pole on the one end of the compound and then greater dipole moment obtained.

### 2.10. Molecular Orbitals and Frontier

Molecular orbitals have a considerable impact on both UV and electrical characteristics [57]. A smaller HOMO–LUMO gap generally indicates greater reactivity compared to a larger energy gap [58]. This energy gap is closely related to the reactivity and stability of the compounds, with a smaller gap suggesting lower kinetic stability and higher chemical reactivity. The calculated energy gap (ΔE) for the studied compounds ranges from 0.097 eV for the more reactive Compound 10 to 0.106 eV for the less reactive Compound 9. Although UV-Vis spectra were not recorded or simulated in the present study, it is expected that such small energy gaps would result in electronic transitions in the UV region (around 250 nm). Future experimental or theoretical UV-Vis studies could further validate these assumptions.

**Figure 9:**
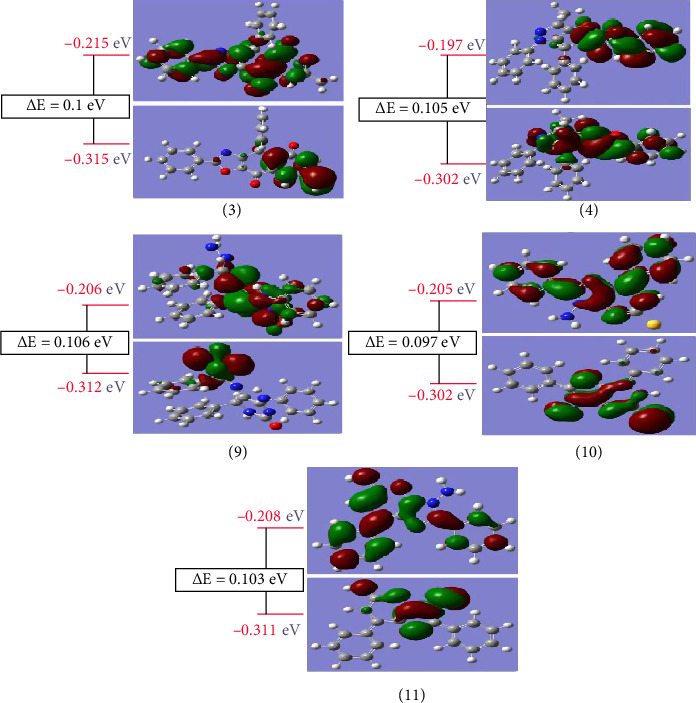
DFT calculations were used to determine the molecular orbital surfaces and energy levels of all substances investigated.

Figure 9 depicts the nodal characteristics of the molecular orbitals for the examined complexes, which show high orbital delocalization, strong orbital overlap, and a small number of nodal planes.  Table 5 shows that the energy gap (ΔE) for examined compounds changes depending on the kind of substitutions. Thione compounds have a greater energy gap than Compounds 9 and 10, suggesting that they are more reactive. Figure 9 shows isodensity surface plots of HOMO and LUMO for the examined substances. Molecules having a wide HOMO–LUMO gap are termed hard; those with a smaller gap are called soft [59].  Table 5 shows the values for *η* and ΔE (HOMO–LUMO). Thione compounds are softer molecules, with *η* ranging from 0.053 for Compound 9 to 0.048 for Compound 10, which is softer than both Compound 9 and the other compounds. ΔE indicates that electronic transitions are more accessible. Quantum chemical characteristics, including global softness (S), electronegativity (χ), absolute softness (σ), chemical potential (π), global electrophilicity (ω), and extra electronic charge (ΔNmax), were determined for each molecule. Compound 10 is an absolute soft molecule with σ = 20.619 eV, while Compound 9 is categorized as hard with σ = 18.868 eV.

As shown in Figure 9, all compounds contain similar nuclei and comparable substations with varied geometrical structures, which play a major role in the modification of different attributes, and therefore, these compounds are separated into distinct portions based on structure. In case of Compound 3, there are five parts as listed in Table 6, and the major percentage is localized on the diazine ring with 90.6% in HOMO state, while in LUMO state, there is delocalization of the electron density with spreading of electron density with different portions over all atoms except one phenyl ring with 0.00%. In Compound 4, there are five parts, the most percentage localized on oxazole ring with 48.9% and one phenyl group with 37.4% in HOMO state, while in LUMO state, the electron density spreading over diazole ring with 24.8%, oxazole ring with 28.7%, one phenyl ring with 27.4%, and O21 with 19.1%. In Compound 9, there is a building up of charge density in the HOMO state on N46–N47 only with 94.9%, while in the HOMO state, there is localization of electron density on diazine ring with 72.6%. In Compound 10, the electron density spreads over all atoms in the HOMO state with different portions except the two phenyl rings with 0.00%, and also in the case of the LUMO state, there is a spreading of electron density over all atoms with different portions except N12 and S20 with 0.00%. In the last Compound 11, in the HOMO state, there is a localization of electron density over the atoms of pyran ring with 68.3%, while in the LUMO state, there is delocalization of electron density and spreading of it with different portions over all atoms.

## 3. Experimental

### 3.1. General Information

All chemicals were procured from Sigma-Aldrich (Taufkirchen, Germany), and solvents were obtained from El-Nasr Pharmaceutical Chemicals Company (analytical reagent grade, Egypt). The 2-thiobarbituric acid was acquired from the Central Laboratory of the Health Ministry. All chemicals were used as received without further purification. Melting points were measured with an uncorrected digital Electrothermal IA 9100 Series apparatus (Cole-Parmer, Beacon Road, Stone, Staffordshire, ST15 OSA, UK). Infrared (IR) spectra were recorded using an FT-IR 460 PLUS (KBr disks) over the range of 4000 to 400 cm^−1^. Proton NMR (^1^H NMR) spectra were obtained with a Bruker 400 MHz NMR spectrometer, using tetramethylsilane (TMS) as the internal standard; chemical shifts are reported in δ (ppm). All analyses were conducted at the Regional Center for Mycology & Biotechnology (RCMB), Al-Azhar University, Naser City, Cairo.

### 3.2. Chemistry

#### 3.2.1. 4-Benzylidene-2-phenyl-1,3-oxazol-5(4H)-one (2)

Compound 4-benzylidene-2-phenyl-1,3-oxazol-5(4H)-one (2) was synthesized by refluxing a mixture of N-benzoylglycine (hippuric acid, 0.1 mol, 17.92 g), benzaldehyde (0.1 mol, 10.61 g), acetic anhydride (0.2 mol, 20.41 g), and sodium acetate (0.1 mol, 8.20 g) in glacial acetic acid (30 mL) for 2 h. Upon cooling, a solid product was formed, which was filtered, washed with cold water, dried, and recrystallized from ethanol to give pale-yellow crystals in 81% yield, with a melting point of 166°C. The IR spectrum (KBr) showed characteristic absorption bands at 3080 cm^−1^ (C–H aromatic), 1695 cm^−1^ (C=O), 1632 cm^−1^ (C=N), 1441 cm^−1^ (C=C), 1270 cm^−1^ (C–N), and 1053 cm^−1^ (C–O). The ^1^H NMR spectrum (400 MHz, DMSO-d_6_) revealed signals at δ 6.71–6.73 (d, 1H, J = 8.0 Hz, aromatic H), 7.12–7.27 (t, 3H, J = 8.0 Hz, aromatic H), 7.50–7.58 (m, 5H, aromatic H), 7.79–7.82 (d, 2H, J = 7.2 Hz, aromatic H), and 6.99 (s, 1H, =CH). The ^13^C NMR spectrum (100 MHz, DMSO-d_6_) showed chemical shifts at δ 113.88, 121.16, 125.21, 127.31, 127.60, 129.57, 129.94, 130.16, 132.76, 135.69, 159.28, and 165.88 ppm. Elemental analysis for C_16_H_11_NO_2_ (molecular weight: 249.08) gave C, 77.21%; H, 4.35%; and N, 5.60%, which is in good agreement with the calculated values: C, 77.10%; H, 4.45%; and N, 5.62%.

#### 3.2.2. 2,9-Diphenyl-5,9-dihydro-6H-oxazolo[4′,5':5,6]pyrano[2,3-d]pyrimidine-6,8(7H)-dione (3)

A mixture of 4-benzylidene-2-phenyloxazol-5(4H)-one (0.01 mol), barbituric acid (0.01 mol), sodium acetate (0.01 mol), and glacial acetic acid (30 mL) was refluxed for 4 h. Upon cooling, the resulting solid was filtered, dried, and recrystallized from ethanol to afford Compound 3 as orange crystals in 59% yield with a melting point greater than 300°C. The IR spectrum (KBr) showed characteristic absorption bands at 3271 cm^−1^ (N–H stretching), 1641 cm^−1^ (C=O stretching), 1546 cm^−1^ (C=N stretching), 1453 cm^−1^ (C=C stretching), 1227 cm^−1^ (C–N stretching), and 1059 cm^−1^ (C–O stretching). The ^1^H NMR spectrum (400 MHz, DMSO-d_6_) displayed signals at δ 5.45 (s, 1H, CH), 7.20–7.22 (t, 2H, J = 8.00 Hz, aromatic H), 7.43–7.45 (t, 1H, J = 8.00 Hz, aromatic H), 7.55 (s, 1H, CH), 7.64–7.66 (d, 2H, J = 8.00 Hz, aromatic H), 7.67–6.99 (d, 2H, J = 8.00 Hz, aromatic H), 7.96–7.99 (t, 3H, J = 8.00 Hz, aromatic H), and a singlet at 13.12 ppm (2H, NH). The ^13^C NMR spectrum (100 MHz, DMSO-d_6_) showed resonances at δ 55.39, 113.90, 124.22, 128.47, 128.95, 129.71, 129.85, 136.83, 137.45, 146.18, 149.62, 152.43, 158.12, and 161.91 ppm. Elemental analysis of C_20_H_13_N_3_O_5_ (M.W. = 359.09) gave C, 66.67%; H, 3.58%; and N, 11.59%, which are in good agreement with the calculated values: C, 66.85%; H, 3.65%; and N, 11.69%.

#### 3.2.3. 3-Methyl-1, 6,8-triphenyl-1,8-dihydropyrazolo[3′,4′:5,6]pyrano[3,2-d]oxazole (4)

A mixture of 4-benzylidene-2-phenyloxazol-5(4H)-one (0.01 mol), an N-phenyl pyrazolone derivative (0.01 mol), sodium acetate (0.01 mol), and glacial acetic acid (30 mL) was refluxed for 4 h. Upon cooling, the solid product was filtered, dried, and recrystallized from ethanol to afford Compound 4 as dark crystals in 73% yield with a melting point of 225°C. The IR spectrum (KBr) showed characteristic absorption bands at 2850 cm^−1^ (C–H, methyl), 1581 cm^−1^ (C=N stretching), 1486 cm^−1^ (C=C stretching), 1265 cm^−1^ (C–N stretching), and 1164 cm^−1^ (C–O stretching). The ^1^H NMR spectrum (400 MHz, DMSO-d_6_) exhibited signals at δ 2.48 (s, 3H, CH_3_), 5.45 (s, 1H, CH), 7.41–7.45 (m, 5H, aromatic H), 7.62–7.70 (m, 5H, aromatic H), and 7.94–8.00 (m, 5H, aromatic H). The ^13^C NMR spectrum (100 MHz, DMSO-d_6_) showed resonances at δ 30.28, 55.43, 114.00, 116.99, 118.78, 122.59, 123.68, 125.04, 126.69, 128.55, 129.90, 130.94, 132.87, 134.11, 134.67, 137.39, 148.36, 151.11, 158.26, 159.68, and 161.66 ppm. Elemental analysis for C_26_H_19_N_3_O_2_ (M.W. = 405.15) gave C, 77.04%; H, 4.70%; and N, 10.37%, which are in good agreement with the calculated values: C, 77.02%; H, 4.72%; and N, 10.36%.

#### 3.2.4. (3-Oxo-1-phenyl-3-(phenylamino)prop-1-en-2-yl)benzimidic Acid (5)

A mixture of 4-benzylidene-2-phenyloxazol-5(4H)-one (0.01 mol) and aniline (0.01 mol) in glacial acetic acid (30 mL) was heated under reflux for 4 h. Upon cooling, the resulting solid was filtered, dried, and recrystallized from ethanol to afford Compound 5 as red crystals in 73% yield, with a melting point of 210°C. The IR spectrum (KBr) showed characteristic absorption bands at 3743 cm^−1^ (O–H stretching), 3274 cm^−1^ (N–H stretching), 1644 cm^−1^ (C=O stretching), 1549 cm^−1^ (C=N stretching), 1454 cm^−1^ (C=C stretching), 1232 cm^−1^ (C–N stretching), and 1062 cm^−1^ (C–O stretching). The ^1^H NMR spectrum (400 MHz, DMSO-d_6_) displayed multiplets at δ 6.65–6.73 (5H, aromatic protons), a singlet at 6.80 (1H, CH), multiplets at 7.25–7.34 (5H, aromatic), and 7.49–7.55 (5H, aromatic), along with singlets at 9.22 (1H, NH) and 10.09 ppm (1H, OH). The ^13^C NMR spectrum (100 MHz, DMSO-d_6_) exhibited chemical shifts at δ 113.86, 116.45, 126.33, 127.53, 129.80, 129.96, 130.27, 130.48, 142.57, 149.86, 155.14, and 166.44 ppm. Elemental analysis for C_22_H_18_N_2_O_2_ (M.W. = 342.14) gave C, 77.20%; H, 5.35%; and N, 8.10%, which are in good agreement with the calculated values: C, 77.17%; H, 5.30%; and N, 8.18%.

#### 3.2.5. *N*-(6-Acetyl-5-hydroxy-3-(phenylamino)-[1,1′-biphenyl]-2-yl)benzimidic Acid (6)

A mixture of (−3-oxo-1-phenyl-3-(phenylamino)prop-1-en-2-yl)benzimidic acid (0.01 mol) and acetylacetone (0.01 mol) in sodium ethoxide solution (prepared in 20 mL of ethanol) was heated under reflux for 4 h. After completion of the reaction, the mixture was cooled to room temperature, and the resulting solid was filtered, dried, and recrystallized from ethanol to afford Compound 6 as orange crystals in 65% yield, with a melting point of 230°C. The IR spectrum (KBr) exhibited absorption bands at 3438 cm^−1^ (O–H stretching), 3162 cm^−1^ (N–H stretching), 1677 cm^−1^ (C=O), 1576 cm^−1^ (C=N stretching), 1489 cm^−1^ (C=C stretching), and 1244 cm^−1^ (C–N stretching). The ^1^H NMR spectrum (400 MHz, DMSO-d_6_) displayed a singlet at δ 3.67 (3H, CH_3_), multiplets at 6.68–6.80 (5H, aromatic), a singlet at 6.82 (1H, CH), multiplets at 7.25–7.38 (5H, aromatic), and 7.49–7.55 (5H, aromatic), as well as singlets at 9.22 (1H, NH), 10.05 (1H, OH), and 11.97 ppm (1H, OH). The ^13^C NMR spectrum (100 MHz, DMSO-d_6_) showed signals at δ 30.25, 113.75, 116.42, 125.48, 127.28, 127.53, 129.50, 129.80, 130.48, 132.80, 142.77, 150.04, 154.93, 157.95, 166.44, 167.13, and 202.74 ppm. Elemental analysis for C_27_H_22_N_2_O_3_ (M.W. = 422.16) gave C, 76.75%; H, 5.25%; and N, 6.61%, which is in excellent agreement with the calculated values: C, 76.76%; H, 5.25%; and N, 6.63%.

#### 3.2.6. 6, 8, 9-Triphenyl-9H-purine-2-thiol (7)

A solution of 3-oxo-1-phenyl-3-(phenylamino)prop-1-en-2-yl)benzimidic acid (0.01 mol) and thiourea (0.01 mol) in sodium ethoxide (prepared in 20 mL of ethanol) was refluxed for 4 h. Upon completion, the reaction mixture was cooled to room temperature, and the resulting solid was filtered, dried, and recrystallized from ethanol to yield Compound 7 as yellow crystals with a 55% yield and a melting point of 220°C. The IR spectrum (KBr) showed absorption bands at 3027 cm^−1^ (aromatic C–H), 1601 cm^−1^ (C=N stretching), 1493 cm^−1^ (C=C stretching), and 1216 cm^−1^ (C–N stretching). The ^1^H NMR spectrum (400 MHz, DMSO-d_6_) exhibited signals at δ 6.83–6.86 (d, 2H, J = 7.3 Hz, aromatic protons), 7.18–7.27 (m, 5H, aromatic), 7.37–7.42 (t, 3H, J = 7.2 Hz, aromatic), 7.94–8.09 (m, 5H, aromatic), and a singlet at 13.35 ppm (1H, SH). The ^13^C NMR spectrum (100 MHz, DMSO-d_6_) displayed chemical shifts at δ 114.08, 122.59, 126.69, 126.77, 130.08, 130.94, 134.02, 134.67, 148.54, 151.19, 158.47 (× 2), 159.83, and 161.48 ppm. Elemental analysis of C_23_H_16_N_4_S (M.W. = 380.11) gave C, 72.60%; H, 4.25%; N, 14.70%; and S, 8.42%, which is in good agreement with the calculated values: C, 72.61%; H, 4.24%; N, 14.73%; and S, 8.43%.

#### 3.2.7. 2-((Hydrazineyl(phenyl)methylene)amino)-3-phenylacrylohydrazide (8)

A mixture of 4-benzylidene-2-phenyloxazol-5(4H)-one (0.01 mol) and hydrazine hydrate (0.01 mol) in ethanol (30 mL) was heated under reflux for 2 h. After completion of the reaction, the mixture was cooled to room temperature, and the resulting solid was filtered, dried, and recrystallized from ethanol to afford Compound 8 as yellow crystals in 50% yield, with a melting point of 200°C. The IR spectrum (KBr) displayed absorption bands at 3227 cm^−1^ (NH), 3132 cm^−1^ (NH_2_), and 1685 cm^−1^ (C=O). The ^1^H NMR spectrum (400 MHz, DMSO-d_6_) showed singlets at δ 3.71 (1H, CH), 3.89 and 4.06 (each 2H, NH_2_), 6.73 (1H, NH), a doublet at 6.86–6.89 (2H, J = 8.00 Hz, aromatic), a triplet at 7.25–7.29 (3H, J = 8.00 Hz, aromatic), a multiplet at 7.49–7.56 (5H, aromatic), and a singlet at 10.10 ppm (2H, NH). The ^13^C NMR spectrum (100 MHz, DMSO-d_6_) exhibited signals at δ 114.03, 115.13, 120.89, 127.29, 127.55, 129.49, 129.95, 130.50, 132.78, 158.10, and 164.70 ppm. Elemental analysis for C_16_H_17_N_5_O (295.35) gave C, 65.10%; H, 5.88%; and N, 23.76%, which is in good agreement with the calculated values: C, 65.07%; H, 5.80%; and N, 23.71%.

#### 3.2.8. 4-Hydrazineyl-5-((hydrazineyl(phenyl)methyl)imino)-6-phenyl-5,6-dihydropyrimidin-2(1H)-one (9)

A mixture of (Z)-3-(((Z)-hydrazineyl(phenyl)methylene)amino)-4-phenylbut-3-enehydrazide (0.01 mol) and urea (0.01 mol) in ethanolic sodium ethoxide (prepared in 30 mL ethanol) was heated under reflux for 4 h. After the reaction was completed, the mixture was cooled, and the precipitated solid was collected by filtration, dried, and recrystallized from ethanol to afford Compound 9 as orange crystals in 68% yield, with a melting point of 220°C. The IR spectrum (KBr) showed absorption bands at 3224 cm^−1^ (N–H stretching), 3130 cm^−1^ (N–H_2_ stretching), and 1677 cm^−1^ (C=O stretching). The ^1^H NMR spectrum (400 MHz, DMSO-d_6_) exhibited signals at δ 3.67 (s, 2H, NH_2_), 3.91 (s, 2H, NH_2_), 4.59 (s, 1H, CH), 4.98 (s, 1H, NH), 5.30 (s, 1H, CH), 5.80 (s, 1H, NH), 7.06–7.08 (d, 2H, J = 8.00 Hz, aromatic), 7.25–7.28 (t, 3H, J = 8.00 Hz, aromatic), 7.50–7.56 (m, 5H, aromatic), and 9.60 (s, 1H, NH). The ^13^C NMR spectrum (100 MHz, DMSO-d_6_) displayed chemical shifts at δ 54.96, 83.55, 127.25, 127.52, 128.06, 129.40, 129.76, 126.96, 130.12, 132.80, 134.97, 135.76, 150.07, 154.88, 158.06, and 165.70 ppm. Elemental analysis for C_17_H_19_N_7_O (M.W. = 337.39) calculated as C, 60.52%; H, 5.68%; and N, 29.06% and found as C, 60.49%; H, 5.64%; and N, 29.10%.

#### 3.2.9. 9-Amino-6,8-diphenyl-1,9-dihydro-2*H*-purine-2-thione (10)

A mixture of 4-hydrazineyl-5-((hydrazineyl(phenyl)methyl)imino)-6-phenyl-5,6-dihydropyrimidin-2(1H)-one (0.01 mol) and thiourea (0.01 mol) in sodium hydroxide and ethanol (30 mL) was heated under reflux for 12 h. Upon cooling, the precipitated solid was collected by filtration, dried, and recrystallized from ethanol to afford Compound 10 as buff-colored crystals in 50% yield with a melting point of 235°C. The IR spectrum (KBr) exhibited absorption bands at 3470 cm^−1^ (N–H stretching), 3368 cm^−1^ (NH_2_ stretching), 1557 cm^−1^ (C=N stretching), 1481 cm^−1^ (C=C stretching), 1296 cm^−1^ (C–N stretching), and 1240 cm^−1^ (C=S stretching). The ^1^H NMR spectrum (400 MHz, DMSO-d_6_) displayed signals at δ 4.34 (s, 2H, NH_2_), 7.25–7.34 (m, 5H, aromatic), 7.47–7.54 (m, 5H, aromatic), and 9.92 (s, 1H, NH). The ^13^C NMR spectrum (100 MHz, DMSO-d_6_) showed chemical shifts at δ 114.02, 127.26, 127.51, 129.44, 129.51, 129.80, 130.02, 132.77, 134.53, 149.93, 154.98, 157.95, and 180.66 ppm. Elemental analysis for C_17_H_13_N_5_S (319.39) calculated as C, 63.93%; H, 4.10%; N, 21.93%; and S, 10.04% and found as C, 63.92%; H, 4.11%; N, 21.92%; and S, 10.03%.

#### 3.2.10. 5-Imino-2,7-diphenylpyrano[2,3-*d*]imidazol-3(5*H*)-amine (11)

A mixture of hydrazineyl-5-((hydrazineyl(phenyl)methyl)imino)-6-phenyl-5,6-dihydropyrimidin-2(1H)-one (0.01 mol) and acetonitrile (0.01 mol) in ethanolic sodium ethoxide (prepared in 30 mL ethanol) was heated under reflux for 2 h. After completion of the reaction, the mixture was allowed to cool, and the formed solid was collected by filtration, dried, and recrystallized from ethanol to yield compound 11 as buff-colored crystals in 73% yield, with a melting point of 220°C. The IR spectrum (KBr) showed absorption bands at 3329 cm^−1^ (N–H stretching), 3197 cm^−1^ (NH_2_ stretching), 1594 cm^−1^ (C=N stretching), 1543 cm^−1^ (C=C stretching), and 1252 cm^−1^ (C–N stretching). The ^1^H NMR spectrum (400 MHz, DMSO-d_6_) exhibited signals at δ 5.85 (s, 2H, NH_2_), 6.71 (s, 1H, CH), 7.26–7.32 (m, 5H, aromatic), 7.49–7.55 (m, 5H, aromatic), and 10.31 (s, 1H, NH). The ^13^C NMR spectrum (100 MHz, DMSO-d_6_) displayed chemical shifts at δ 54.96, 113.73, 115.11, 127.26, 127.52, 129.42, 129.77, 129.97, 131.64, 132.79, 138.15, 149.86, 154.89, 157.93, and 165.77 ppm. Elemental analysis for C_18_H_14_N_4_O (M.W. = 302.34) calculated as C, 71.51%; H, 4.67%; N, 18.53%; and O, 5.29% and found as C, 71.50%; H, 4.68%; and N, 18.53%.

### 3.3. ADMET Properties

Using the internet web server SwissADME (http://www.swissadme.ch/), the pharmacokinetic characteristics, such as polar surface area and AlogP, were examined. Pk, the absorption, distribution, metabolism, excretion, and toxicity of the ligands were assessed using the CSM ADMET web server [60]. Drug absorption qualities were determined using CaCO_2_ permeability, intestinal absorption, skin permeability, and interaction with P-glycoprotein substrates or inhibitors. A drug's distribution is regulated by its permeability across the BBB, CNS penetration, and volume of distribution (VD). The Cytochrome P450 model was used to assess drug metabolism, which contains CYP2D6 and CYP3A4 substrates as well as CYP1A2, CYP2C19, CYP2C9, CYP2D6, and CYP3A4 inhibitors. The total clearance model was used to describe drug excretion, and interactions with renal OCT2 substrates were assessed.

### 3.4. Preparation of Protein

The three-dimensional crystal structure of the SARS-CoV-2 main protease (Mpro) in complex with the N3 inhibitor (PDB ID: 6LU7, resolution: 2.16 Å) was obtained from the Protein Data Bank (https://www.rcsb.org) [61]. According to Rosidi et al., the PDB serves as a global archive for the three-dimensional structural data of biomacromolecules. The 6LU7 structure consists of two chains: Chain A representing the protease enzyme and Chain C representing the co-crystallized ligand N3. Protein preparation was carried out using MOE software (Version 2022) [12, 25, 28, 62–65]. Initially, the co-crystallized ligand (N3) and water molecules were removed. Hydrogen atoms were added using standard geometry, partial charges were automatically assigned, and any structural errors or missing atoms were corrected. The potential binding pocket was identified using the Alpha Site Finder tool in MOE, which generated dummy atoms around the predicted active site based on alpha spheres. Molecular docking was conducted to investigate the binding behavior of the test ligands with the protease active site. A rigid receptor flexible ligand approach was applied, in which the protease was kept rigid while the ligands were treated as fully flexible during docking. Ligand placement was performed using the Triangle Matcher algorithm, and preliminary scoring was done using the London dG scoring function. Final pose refinement and rescoring were carried out using the GBVI/WSA dG scoring function for more accurate binding energy estimation. Ligand structures were constructed using ChemDraw 12.0 and further processed and minimized in MOE using the MMFF94 force field until convergence at an RMSD gradient of 0.1 kcal·mol^−1^·Å^−1^ was reached. Binding affinities were evaluated based on the docking score (S, in kcal/mol), where more negative values represent stronger interactions [66].

## 4. Conclusion

The present study successfully identified novel pyrimidine-based derivatives with promising inhibitory activity against the SARS-CoV-2 main protease (Mpro). Among the synthesized compounds, derivatives 9, 4, and 11 exhibited the most favorable binding affinities in molecular docking analyses, involving key interactions within the active site. Additionally, DFT calculations and ADME profiling supported their potential as effective antiviral candidates. These findings provide a solid foundation for further biological evaluation and structure optimization toward the development of novel COVID-19 therapeutics.

## Figures and Tables

**Table 1:** Using Lipinski's rule of five (RO5) of Mpro of COVID-19 and selected bioactive components of produced compounds as potential protein inhibitors.

Ligands	Molecular formula	Molecular weight < 500	LogP < 5	H-bond donor < 5	H-bond acceptor < 10	Violations	Meet RO5 criteria
N3	C_35_H_48_N_6_O_8_	680.00	0.6967	5	12	+	−
3	C_21_H_13_N_3_O_3_S	374.00	−0.797240	0	3	−	−
4	C_26_H_19_N_3_O_2_	386.00	−1.098180	0	2	−	−
9	C_23_H_22_N_6_O	376.00	−2.053570	0	1	−	−
10	C_17_H_13_N_5_S	306.00	−1.121820	0	0	−	−
11	C_18_H_14_N_4_O	288.00	−1.273430	0	2	−	−

**Table 2:** The pharmacokinetics of a subset of the bioactive chemicals of synthesized compounds as determined by the SwissADME and pkCSM ADMET web servers.

Parameters	N3	3	4	9	10	11
PSA	197.83	123.84	53.08	103.90	104.61	80.83
Absorption						
Water solubility	−4.144	−3.749	−3.248	−3.197	−2.902	−2.892
CaCo_2_ permeability	0.639	0.394	1.027	0.824	0.769	0.82
Intestinal absorption	57.884	94.791	98.833	83.862	86.061	83.666
Skin permeability	−2.734	−2.737	−2.735	−2.735	−2.735	−2.735
P-glycoprotein substrate	+	+	−	+	+	+
P-glycoprotein-I inhibitor	+	+	−	+	+	+
P-glycoprotein-II inhibitor	+	+	+	+	+	+
Distribution						
VDss (human)	−0.762	−0.154	−0.322	0.015	0.194	−0.064
Fraction unbound	0.067	0.191	0.351	0.172	0.162	0.272
BBB permeability	−1.261	−0.182	0.714	−0.973	−0.763	0.501
CNS permeability	−3.568	−1.883	−1.453	−2.02	−1.93	−1.864
Metabolism						
CYP2D6 substrate	−	−	−	+	−	−
CYP3A4 substrate	+	+	+	+	+	+
CYP1A2 inhibitor	−	+	−	+	+	+
CYP2C19 inhibitor	−	+	+	+	+	+
CYP2C9 inhibitor	−	+	+	+	+	+
CYP2D6 inhibitor	−	−	−	−	+	+
CYP3A4 inhibitor	+	+	+	+	−	+
Excretion						
Total clearance	0.653	−0.021	0.851	0.502	0.47	0.447
Renal OCT2 substrate	−	−	−	+	+	+
Toxicity						
AMES toxicity	−	−	−	−	+	+
Maximum tolerated dose	−0.348	0.523	0.537	1.071	0.205	−0.113
hERG I inhibitor	−	−	−	−	−	−
hERG II inhibitor	+	+	+	+	+	+
Oral rat acute toxicity (LD50)	3.63	3.397	2.298	2.301	2.515	2.351
Oral rat chronic toxicity (LOAEL)	3.935	0.513	−0.134	2.248	1.342	0.963
Hepatotoxicity	+	−	+	−	−	−
Skin sensitization	−	−	−	−	−	−
T. pyriformis toxicity	0.285	0.286	0.285	0.286	0.285	0.285
Minnow toxicity	4.136	2.981	−1.255	0.478	1.234	1.697

**Table 3:** The binding scores, RMSD values, distance measurements, and receptor interactions of the most promising compounds (3, 4, 9, 10, and 11) were evaluated in comparison to reference N3 for their potential efficacy against COVID-19.

Comp.	Score (kcal/mol)	RMSD	Receptor interactions	Distance (Å)	*E* (kcal/mol)
N3	−15.2100	1.8721	Met49 (H-donor)	3.11	−0.90
			Met49 (H-donor)	3.94	−0.70
			Met49 (H-donor)	4.25	−1.80
			Thr190 (H-donor)	3.03	−2.00
			Asn142 (H-acceptor)	2.95	−1.50
			His41 (H-acceptor)	3.05	−0.50
			Cys145 (H-acceptor)	4.22	−5.4
			Glu166 (H-acceptor)	3.59	−10.20

3	−11.5558	1.8025	Pro52 (H-acceptor)	3.99	−0.50
			Gly143 (pi-H)	3.81	−1.00

4	−12.3842	1.5425	Leu167 (pi-H)	4.70	−0.70
			Thr190 (pi-H)	4.04	−0.80
			His41 (pi-H)	3.92	0.00

9	−12.7032	1.8003	Asn142 (pi-H)	4.40	−0.50

10	−10.5335	1.8359	Leu141 (H-donor)	3.35	−1.00
			His163 (H-acceptor)	3.74	−2.20

11	−12.1338	1.1915	Glu166 (pi-H)	4.40	−3.30

**Table 4:** The Mulliken charges on the donating atoms of the compounds studied were determined using DFT calculations.

(3)		(4)		(9)		(10)		(11)	
N1	−0.216	N6	−0.246	N13	−0.317	N8	−0.324	N8	−0.039
N6	−0.291	N9	−0.299	N15	−0.387	N10	−0.012	N10	−0.295
S7	−0.758								
O9	0.435								

O15	−0.368	O21	−0.268	O18	−0.434	N12	−0.283	N35	−0.287

N22	−0.311	N36	−0.277	N19	−0.239	N15	−0.304	N7	−0.479
O23	−0.197	O38	−0.222	N21	−0.253	N17	−0.183		

				N46	−0.198	S20	−0.726	O2	−0.264
				N47	−0.254				

**Table 5:** DFT calculations were employed to determine various properties of the examined compounds, including energy values (HOMO, LUMO, and energy gap ΔE/eV), hardness (η), global softness (S), electronegativity (χ), absolute softness (σ), chemical potential (μ), global electrophilicity (ω), and extra electronic charge (ΔNmax).

Parameters	Compound 3	Compound 4	Compound 9	Compound 10	Compound 11
HOMO, H	−0.315	−0.302	−0.312	−0.302	−0.311
LUMO, L	−0.215	−0.197	−0.206	−0.205	−0.208
I = −H	0.315	0.302	0.312	0.302	0.311
A = −L	0.215	0.197	0.206	0.205	0.208
ΔE = L − H	0.1	0.105	0.106	0.097	0.103
η=	0.05	0.0525	0.053	0.0485	0.0515
χ = −(H − L/2)	0.265	0.2495	0.259	0.2535	0.2595
σ = 1/η	20	19.048	18.868	20.619	19.417
S = 1/2η	10	9.524	9.434	10.309	9.709
Pi = −χ	−0.265	−0.2495	−0.259	−0.2535	−0.2595
ω = (Pi)^2^/2η	0.7023	0.5929	0.6328	0.6625	0.6538
ΔN_max_ = χ/*η*	5.3	4.7524	4.8868	5.2268	5.0388

**Table 6:** The composition of the frontier molecular orbitals for the studied compounds was analyzed using DFT with the B3LYP functional and the CEP-31G basis set.

	**Compound 3**			**Compound 4**	
	**H (%)**	**L (%)**		**H (%)**	**L (%)**

Diazine ring (N1–N6)	90.6	31.4	Diazole ring (C5–N9)	0.0	24.8
Oxazole ring (C12, C13, N22, O23, C24)	1.8	23.0	Oxazole ring (C22, C23, N36, C37, O38)	48.9	28.7
Phenyl rings (C25–C30)	3.4	0.0	Phenyl rings (C39-C44)	37.4	27.4
(C16–C21)	0.0	12.8	(C25-C30)	0.0	0.0
-S17	4.2	5.6	(C10-C15)	0.0	0.0
C11═O10	0.0	13.2	O21	13.7	19.1
C5═O9	0.0	14.0	-CH3	0.0	0.0
			C1		

	**Compound 9**			**Compound 10**	
	**H (%)**	**L (%)**		**H (%)**	**L (%)**

Diazine ring (C12–C17)	0.0	72.6	Diazine ring (C9, C11, N15–C18)	36.1	23.8
Phenyl rings (C1–C6)	0.0	0.0	Diazole ring (C7–N10)	31.7	24.5
(C35-C40)	4.1	0.0			
(C22-C28)	0.0	8.7			
N19	0.0	4.8	Phenyl rings (C1–C6)	0.0	24.4
N21	0.0	5.8	(C21–C26)	0.0	27.3
N46-N47	94.9	0.0	-N12	16.2	0.0
O18	0.0	8.1	-S20	16.0	0.0

	**Compound 11**				
	**H (%)**	**L (%)**	**H**	**L (%)**	

Pyrane ring (C1–C6)	68.3	28.8	N7	8.4	
			N35	7.9	
Phenyl rings (C12–C17)	0.0	31.5	Diazole ring (N8–N10)	15.4	
(C18–C23)	0.0	26.8			

## Data Availability

The data that support the findings of this study are available in the supporting information of this article.

## Supporting Information

It includes the graphical abstract, Tables S1–S5, and comprehensive analysis of the synthesized compounds presented in Supporting Figures 1–31.
