# Supplementary material for: DFT Investigations and Molecular Docking as Potent Inhibitors of SARS-CoV-2 Main Protease of Novel Pyrimidine Dione Derivatives
Source: Biochem Res Int. 2025 Aug 11;2025:7961294. doi: 10.1155/bri/7961294 (PMC12360883; doi:10.1155/bri/7961294)
Supplement: Supporting Information — Additional supporting information is available online in the Supporting Information section. [file 7961294.f1.docx]

**Graphical Abstract: Provided as a supplementary file**


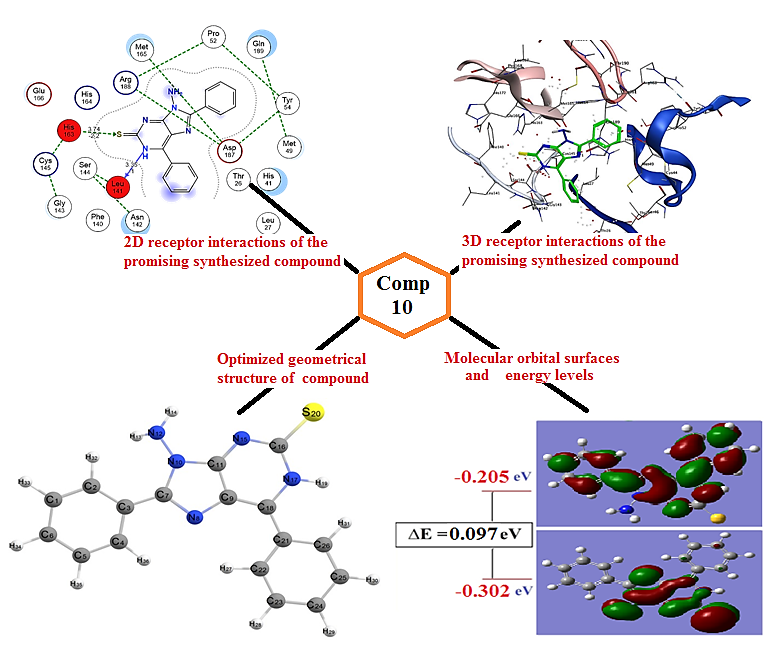


**SPECTRAL ANALYSIS**


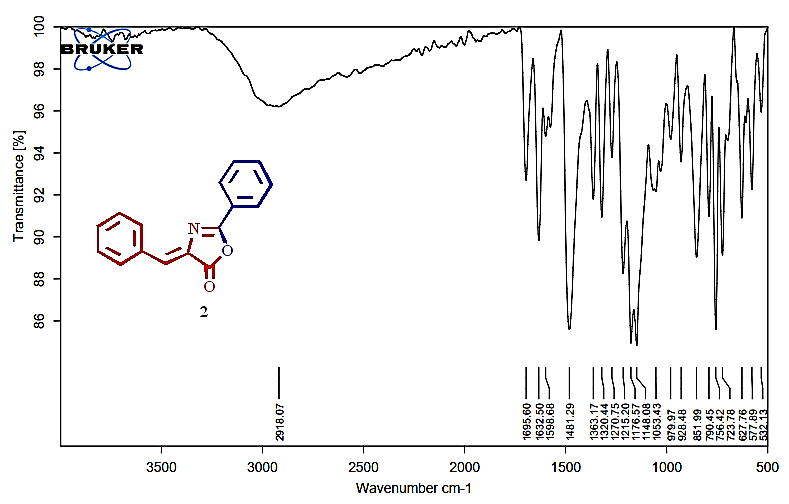


**Fig. 1**: **IR Spectrum of compound 2**


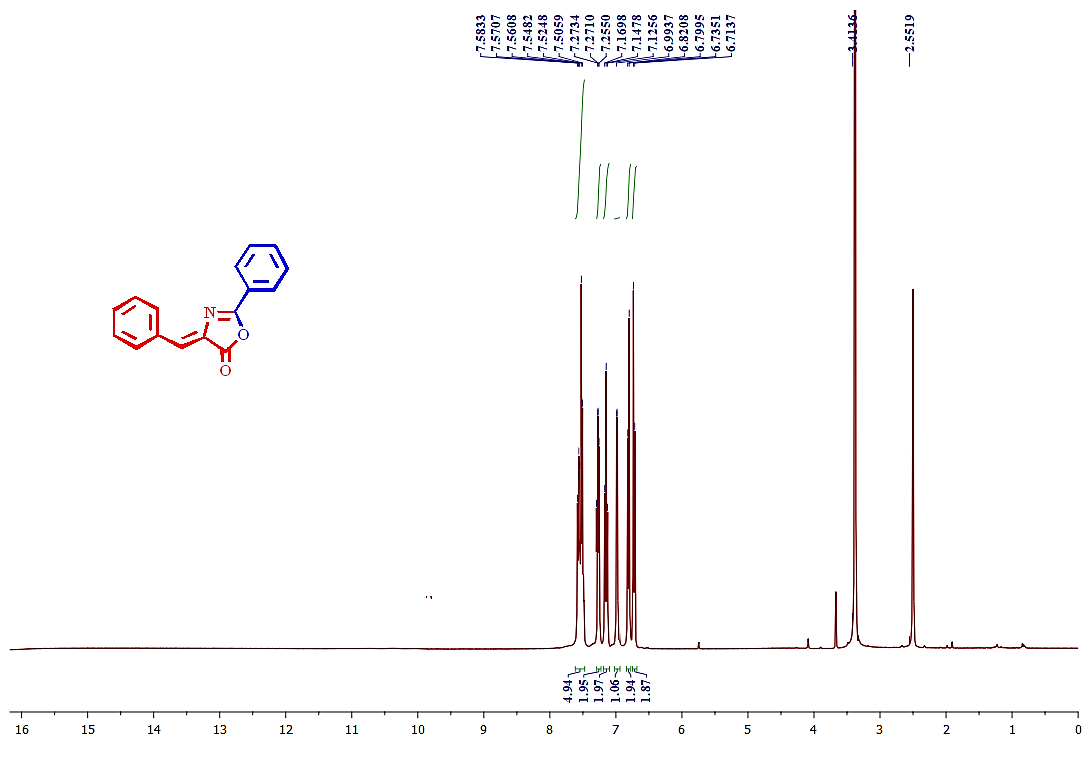


**Fig. 2**: **^1^H-NMR Spectrum of compound 2**

**
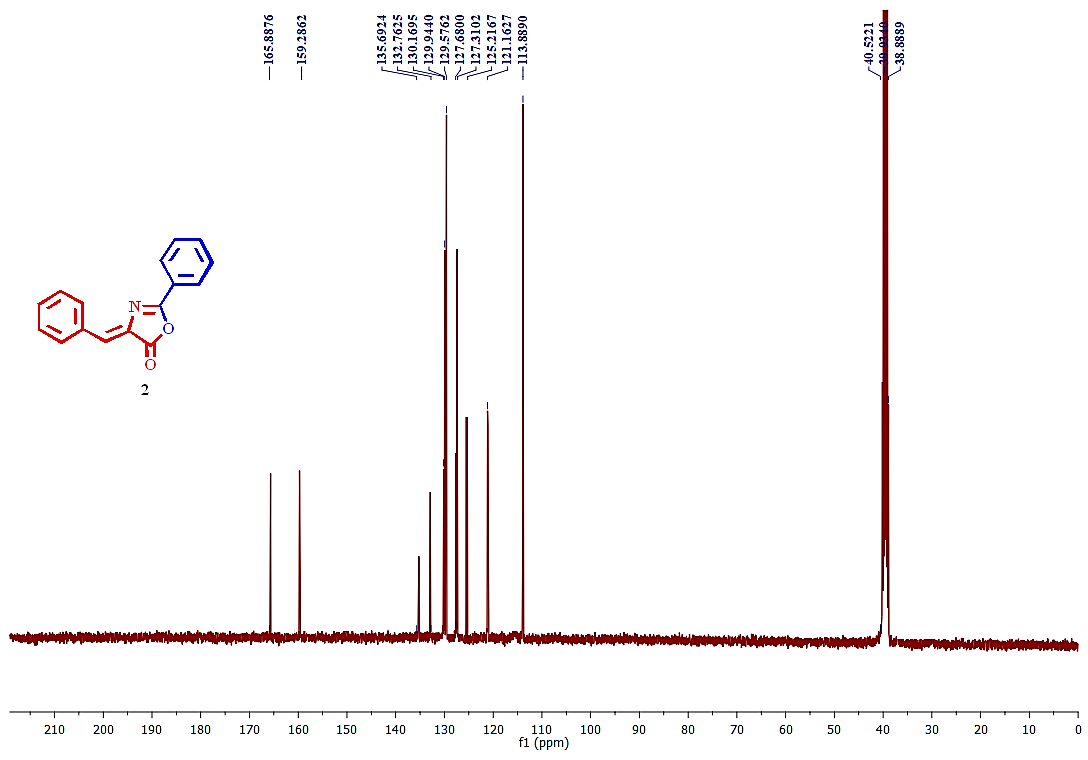
**

**Fig. 3**: **^13^C-NMR Spectrum of compound 2**


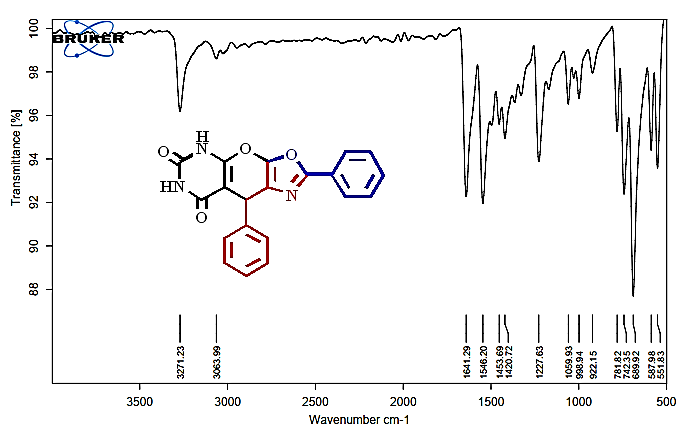


**Fig. 4**: **IR Spectrum of compound 3a**

**
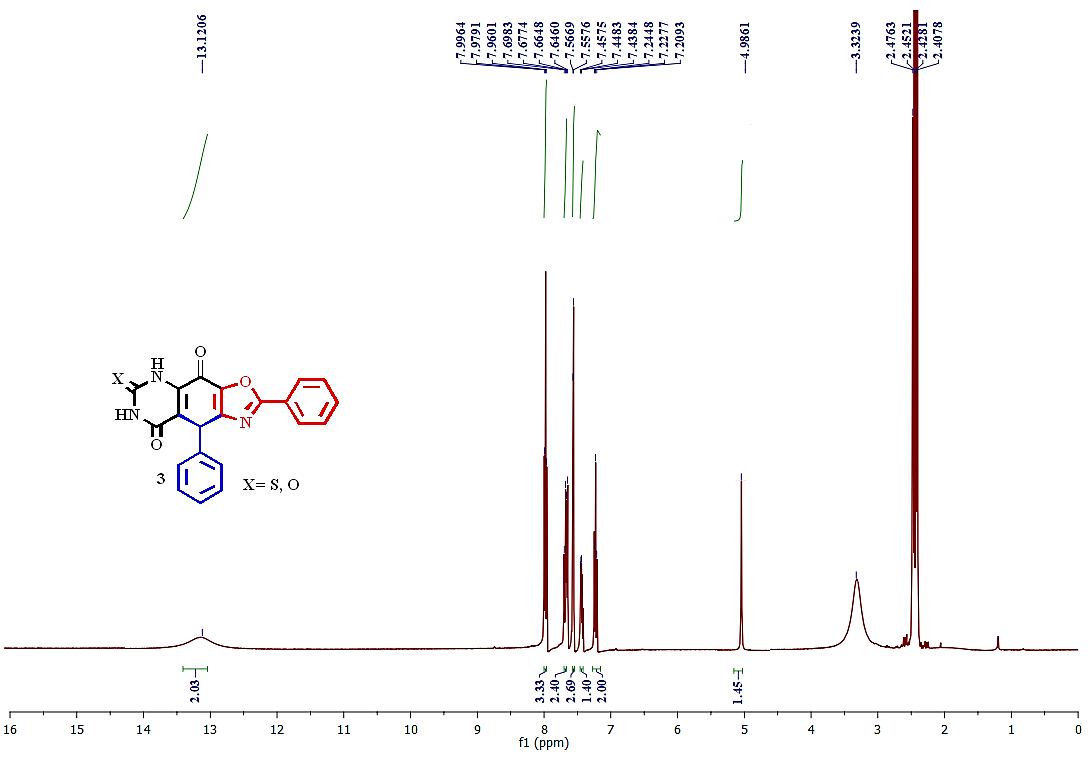
**

**Fig. 5**: **^1^H-NMR Spectrum of compound 3a**

**
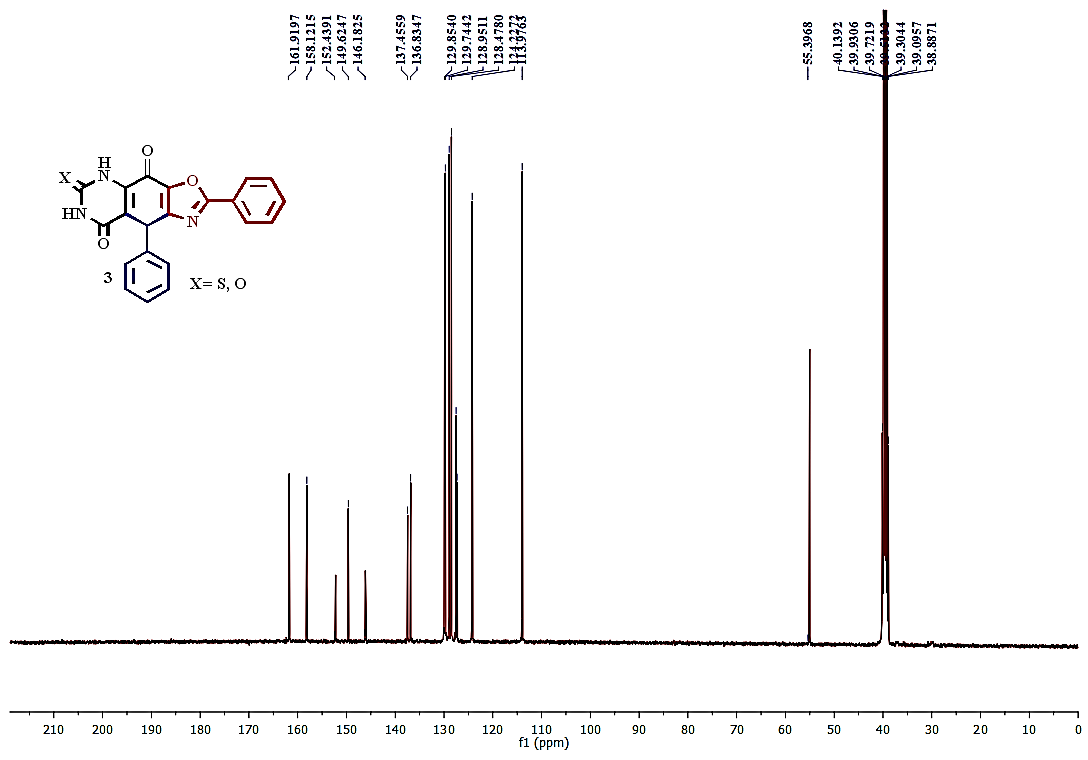
**

**Fig. 6**: **^13^C-NMR Spectrum of compound 3a**

**
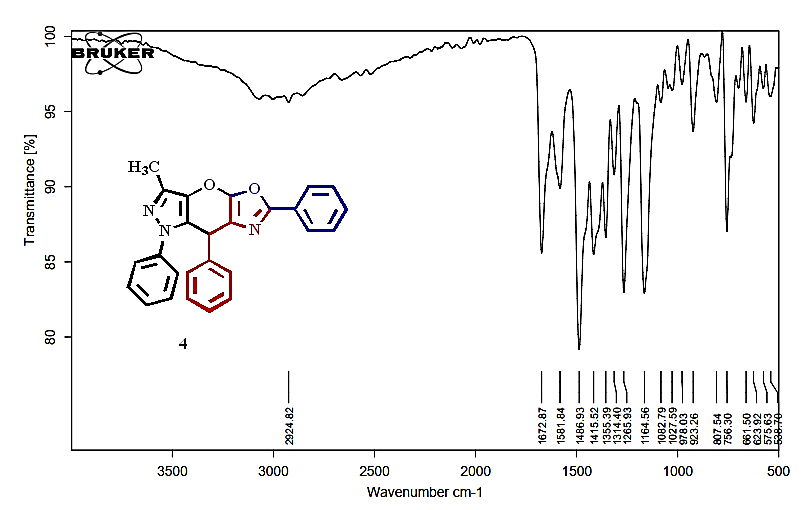
**

**Fig. 7**: **IR Spectrum of compound 4**

**
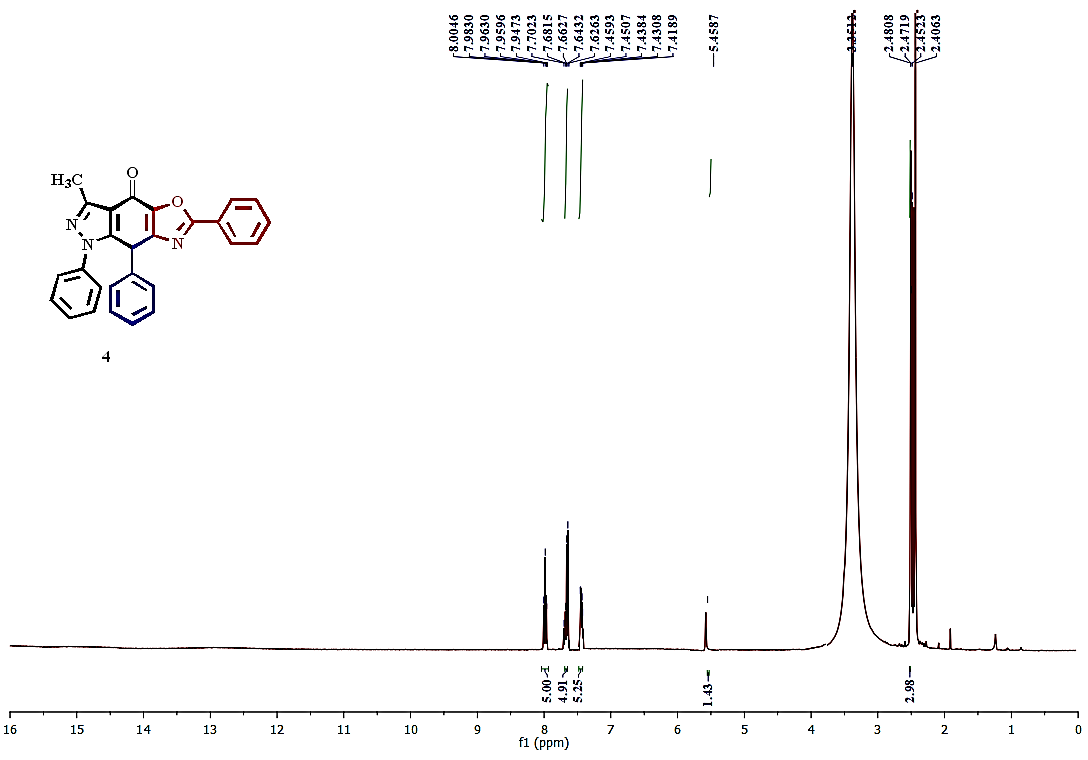
**

**Fig. 8**: **^1^H-NMR Spectrum of compound 4**


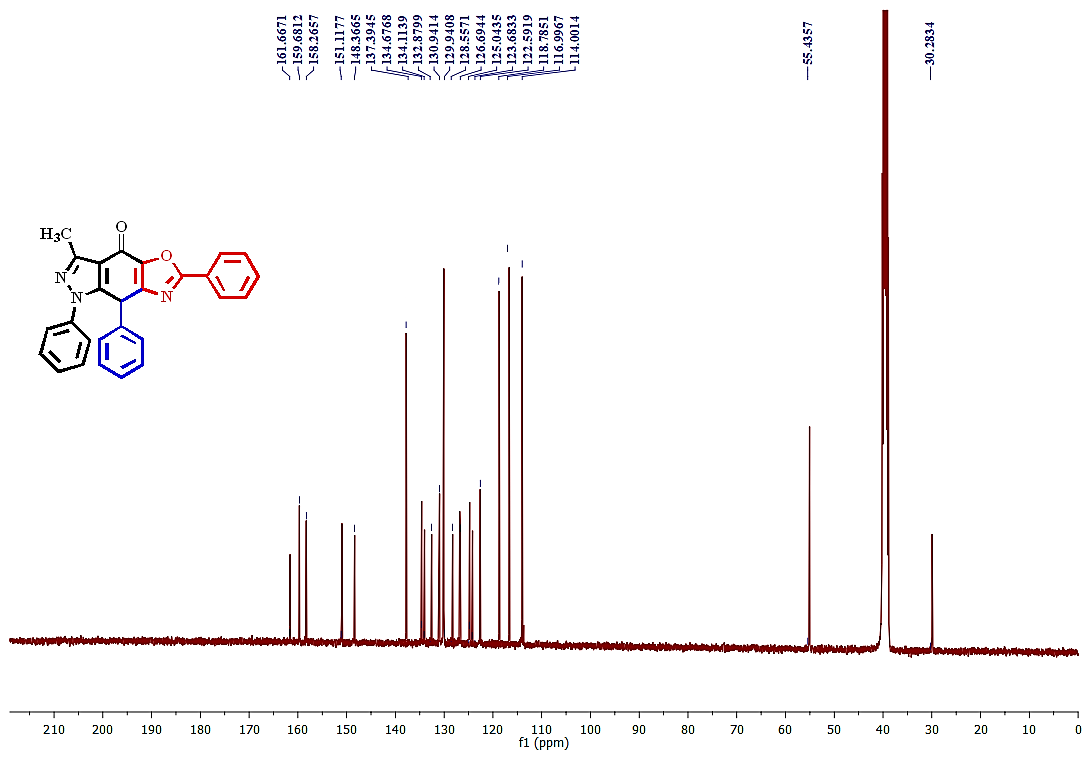


**Fig. 9**: **^13^C-NMR Spectrum of compound 4**

**
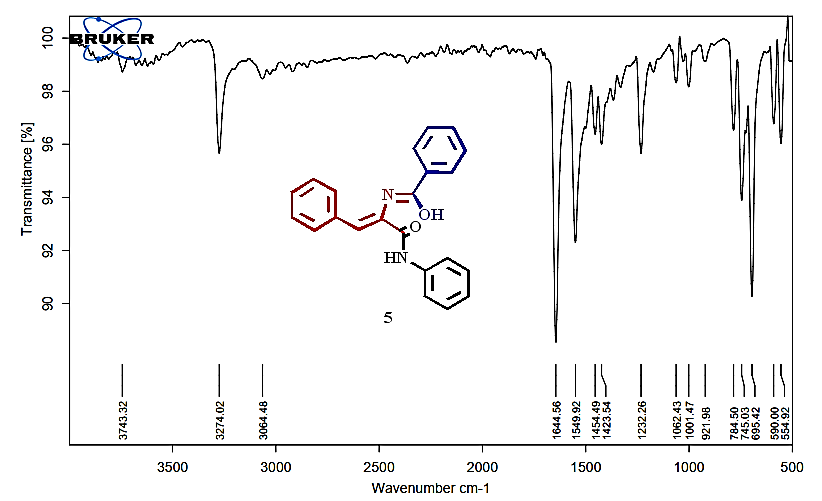
**

**Fig. 10**: **IR Spectrum of compound 5**


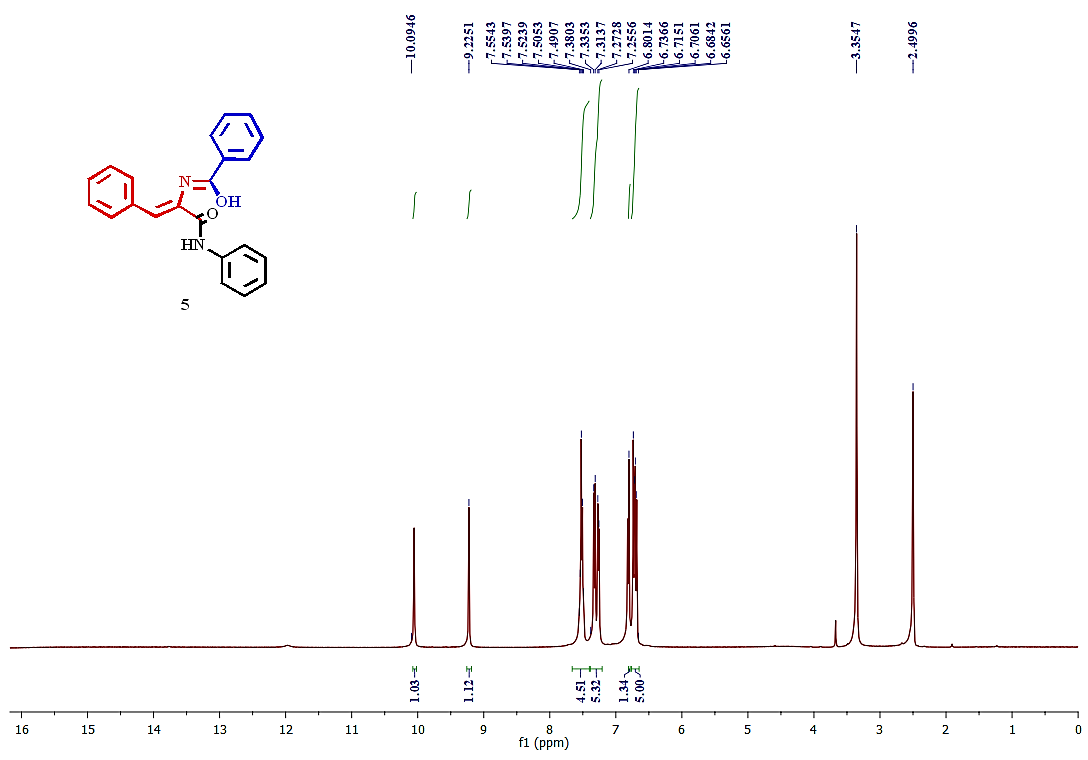


**Fig. 11**: **^1^H-NMR Spectrum of compound 5**


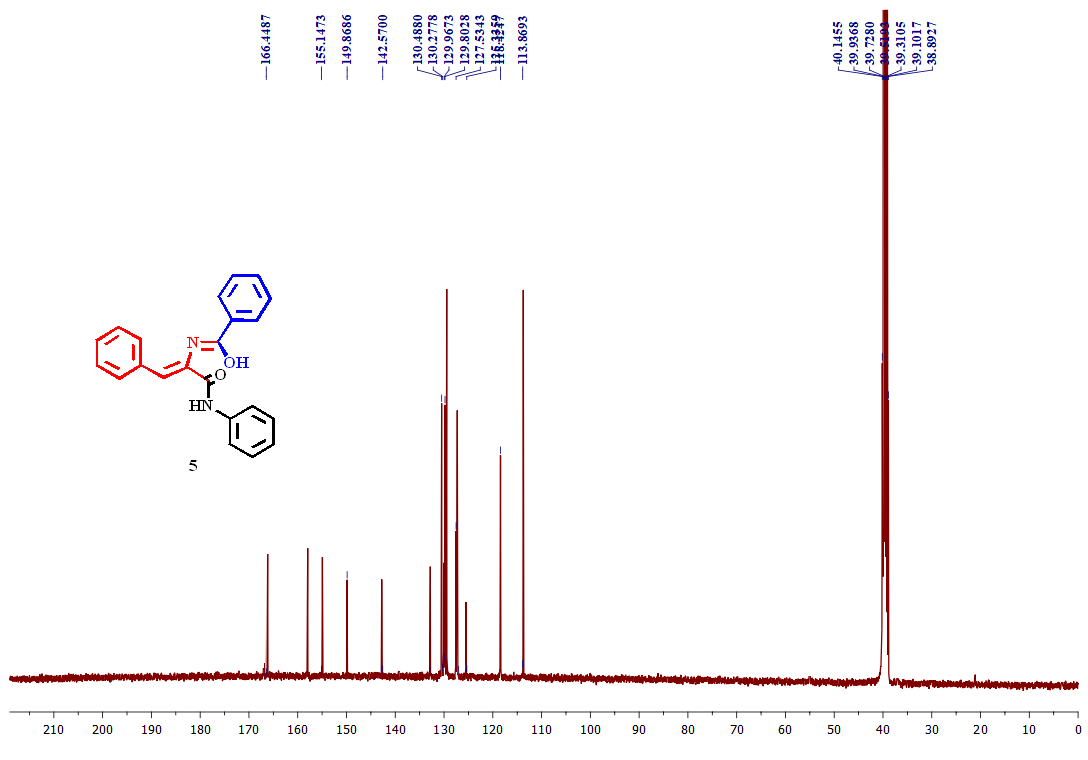


**Fig. 12**: **^13^C-NMR Spectrum of compound 5**


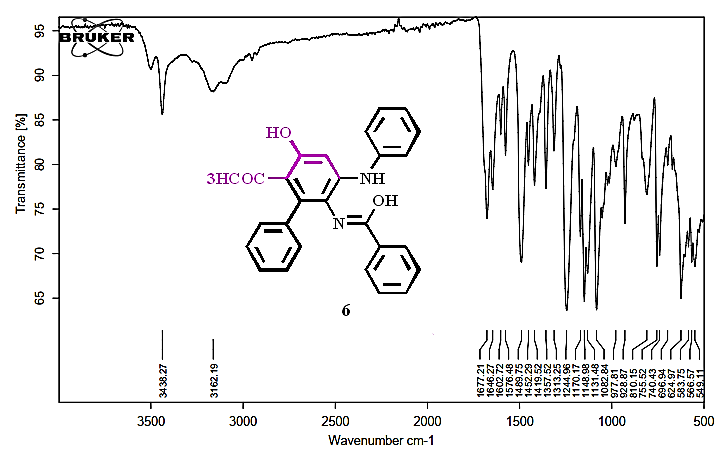


**Fig. 13**: **IR Spectrum of compound 6**


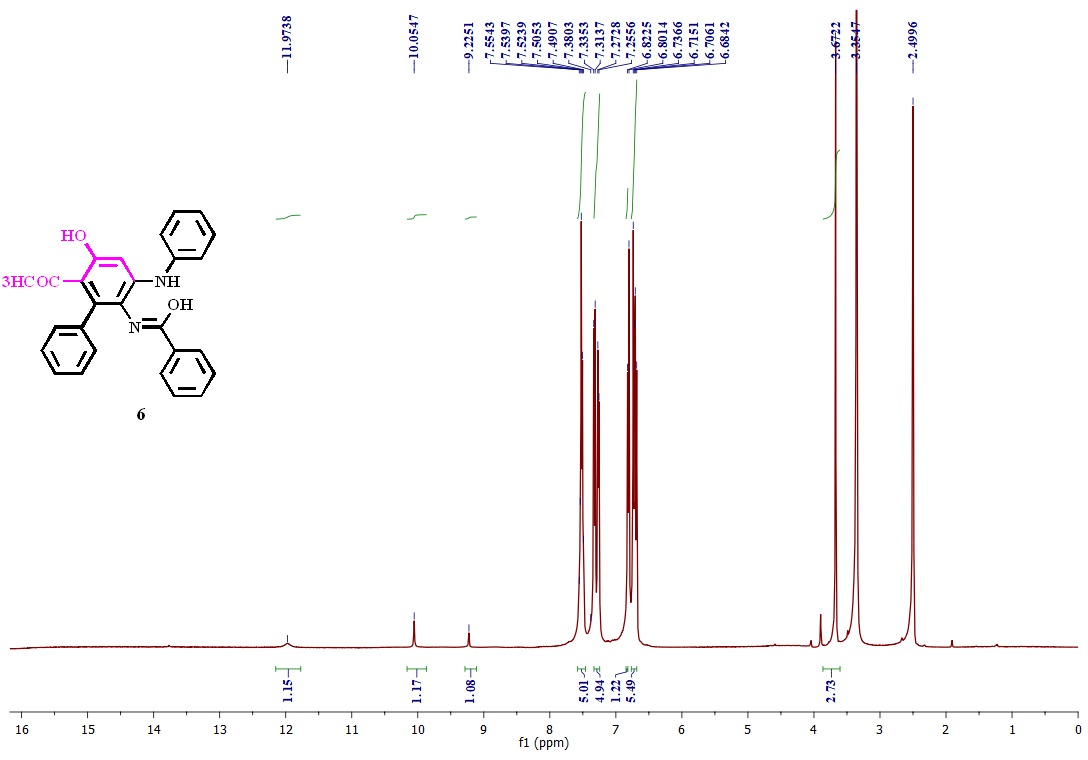


**Fig. 14**: **^1^H-NMR Spectrum of compound 6**


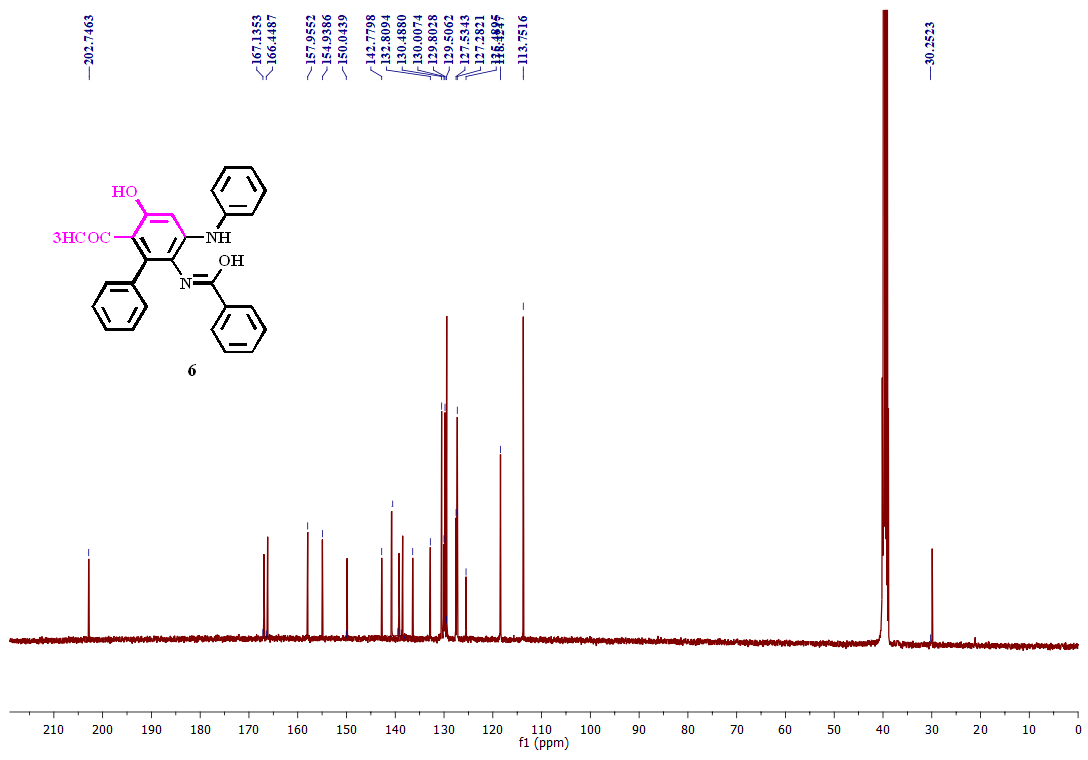


**Fig. 15**: **^13^C-NMR Spectrum of compound 6**


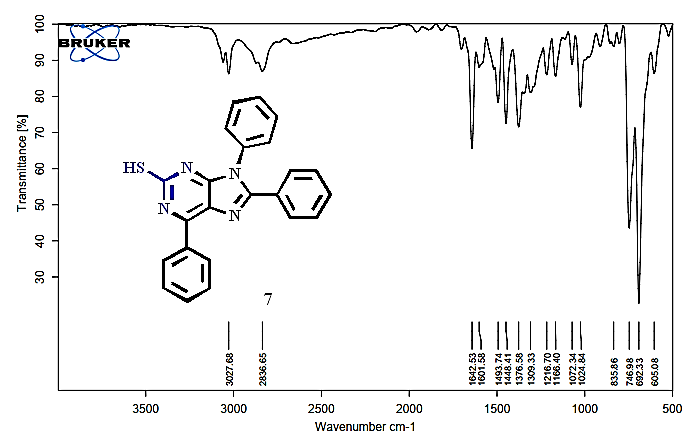


**Fig. 16**: **IR Spectrum of compound 7**


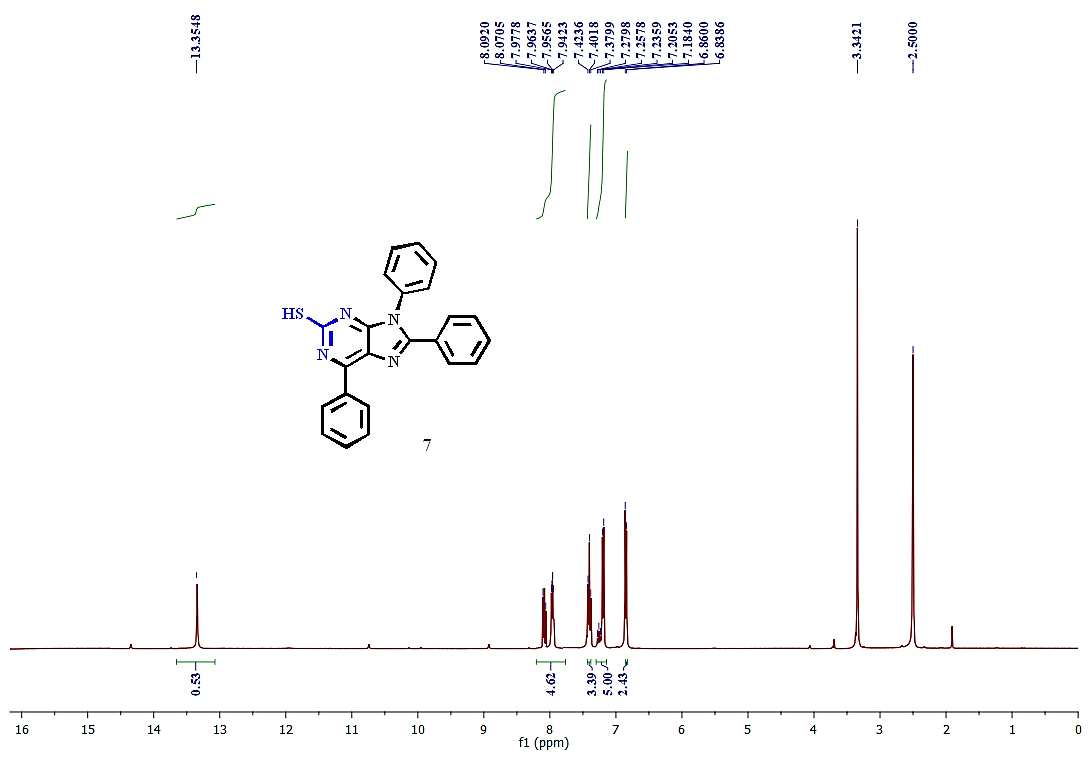


**Fig. 17**: **^1^H-NMR Spectrum of compound 7**


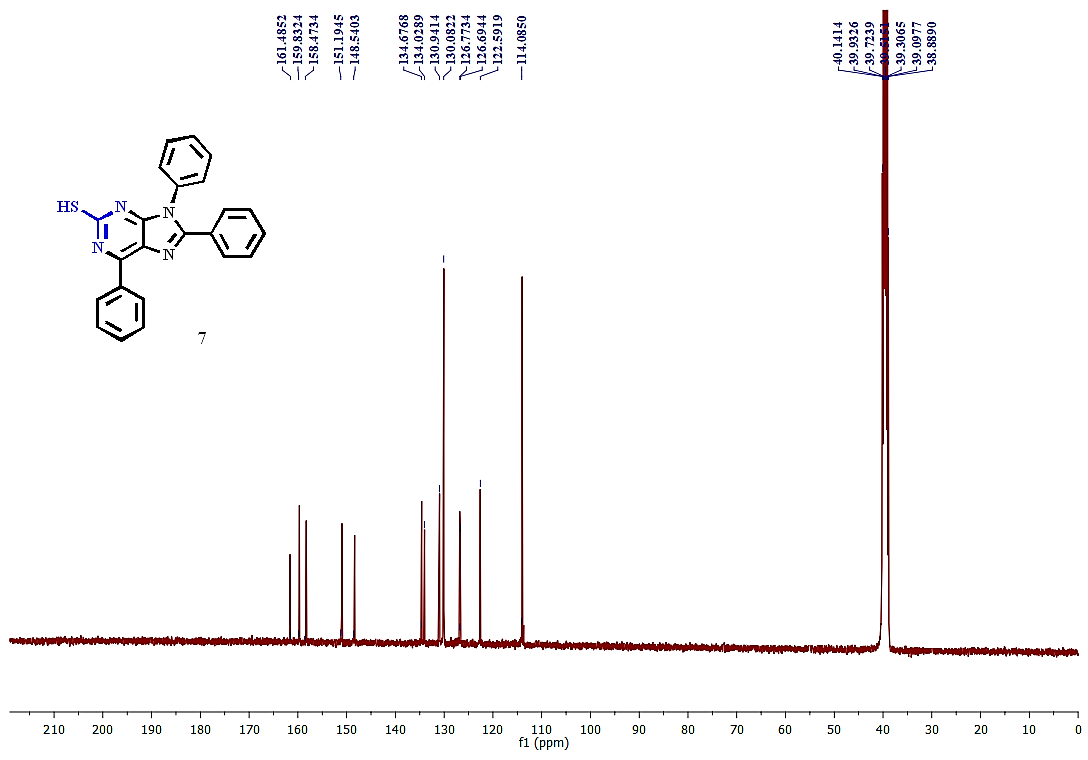


**Fig. 18**: **^13^C-NMR Spectrum of compound 7**


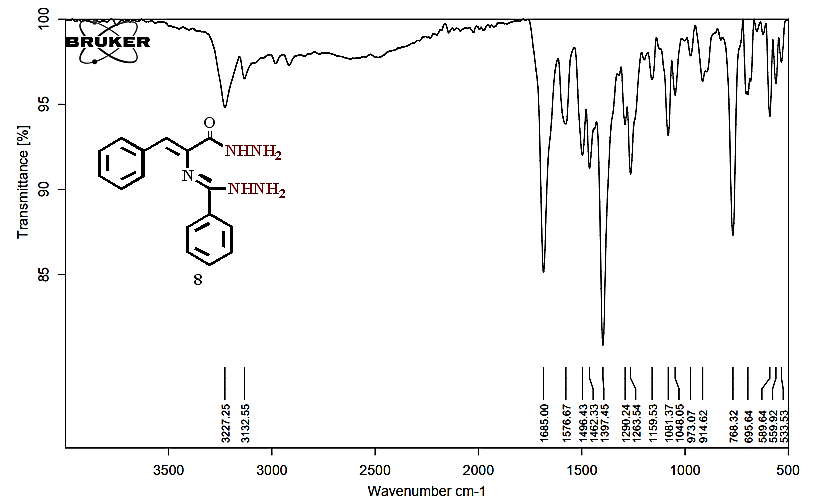


**Fig. 19**: **IR Spectrum of compound 8**

**
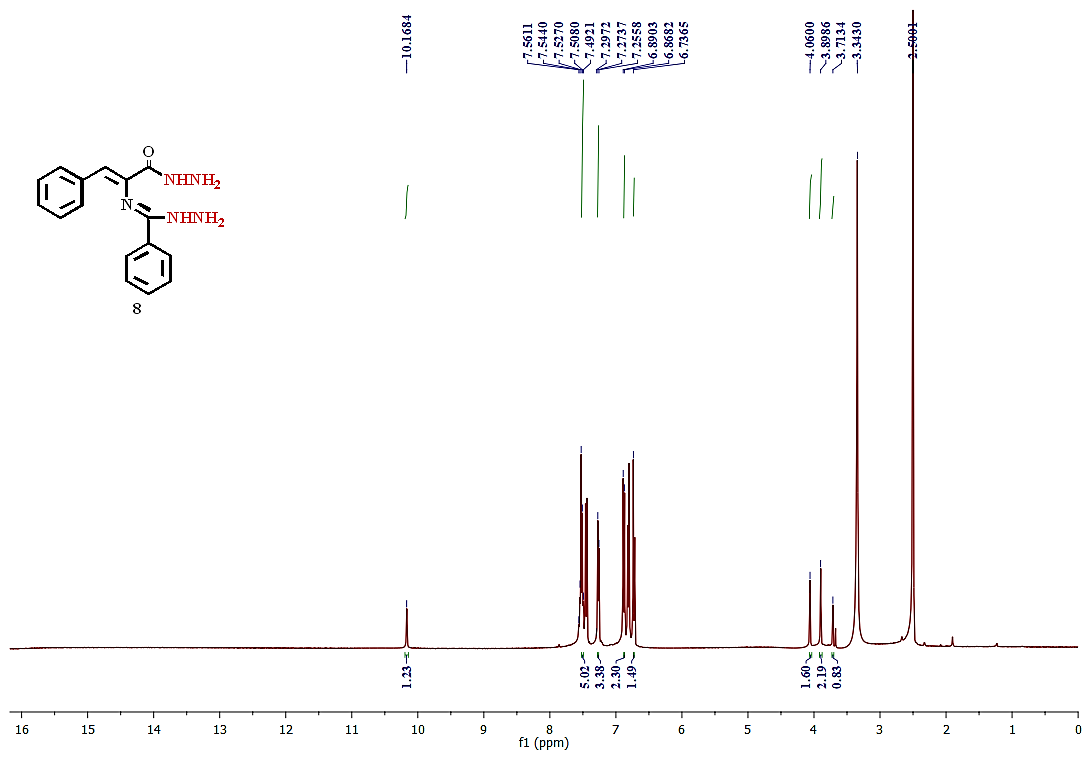
**

**Fig. 20**: **^1^H-NMR Spectrum of compound 8**

**
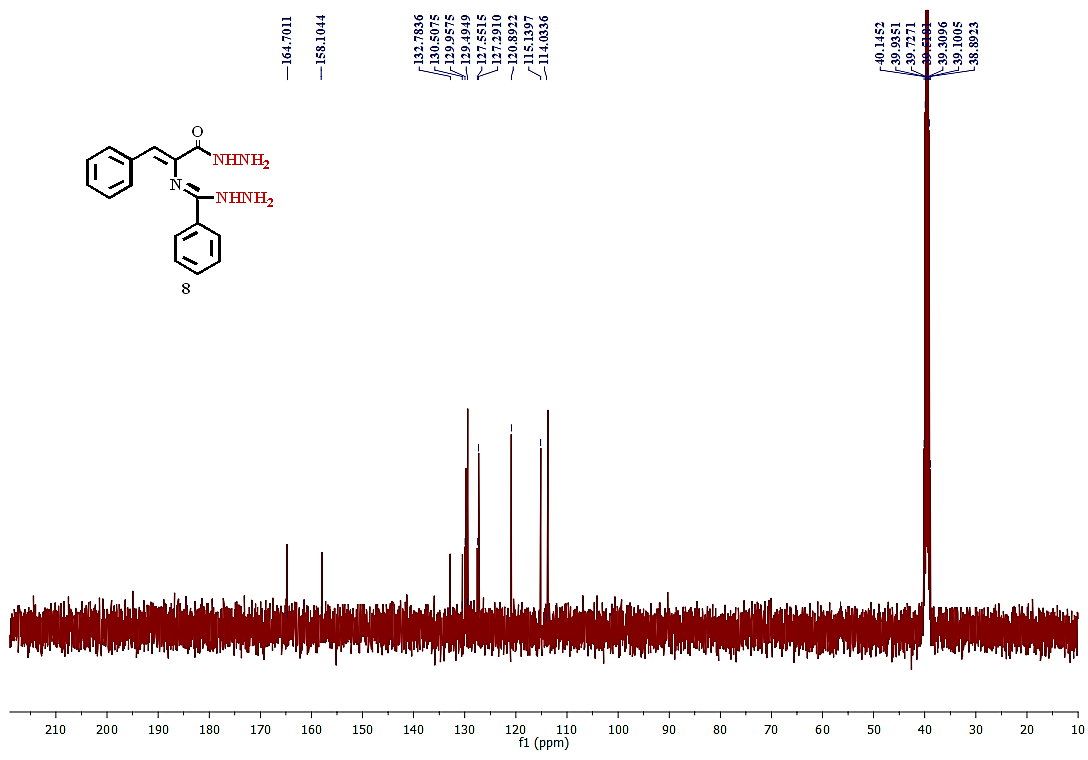
**

**Fig. 21**: **^13^C-NMR Spectrum of compound 8**

**
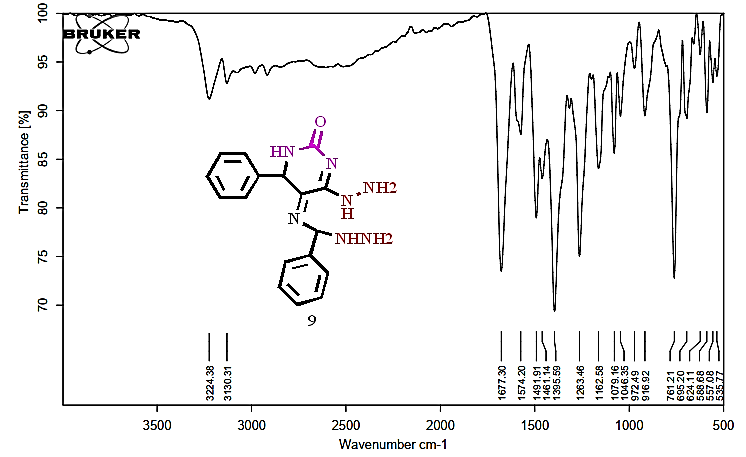
**

**Fig. 22**: **IR Spectrum of compound 9**

**
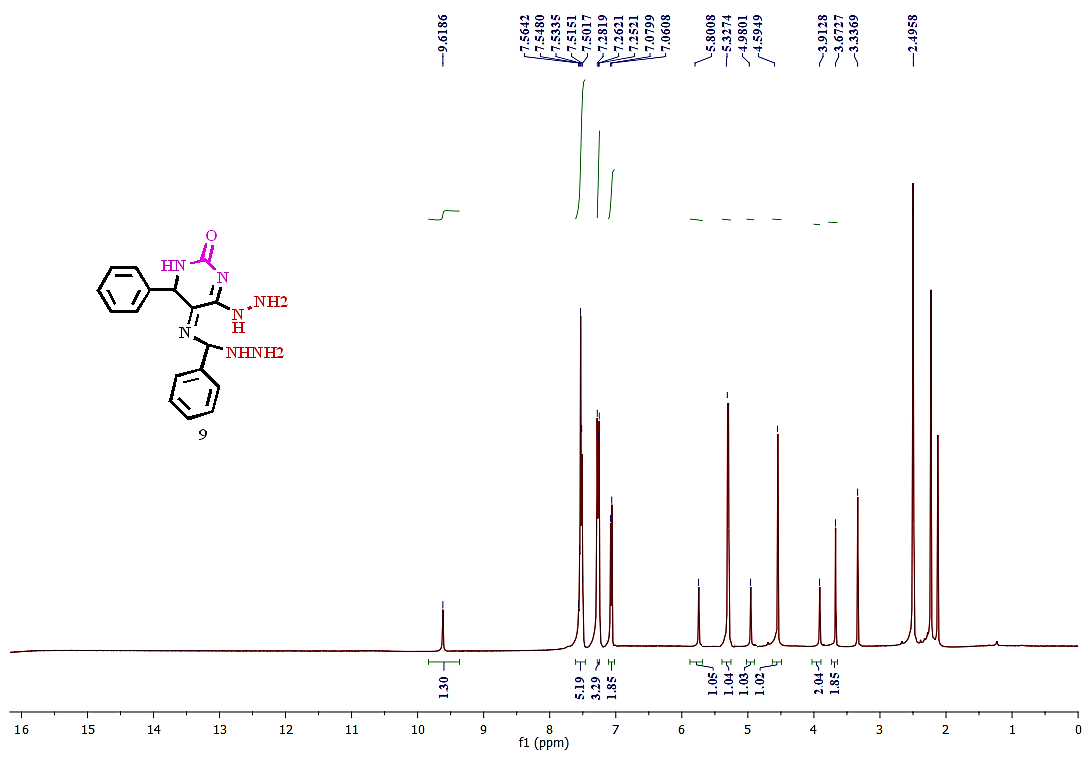
**

**Fig. 23**: **^1^H-NMR Spectrum of compound 9**

**
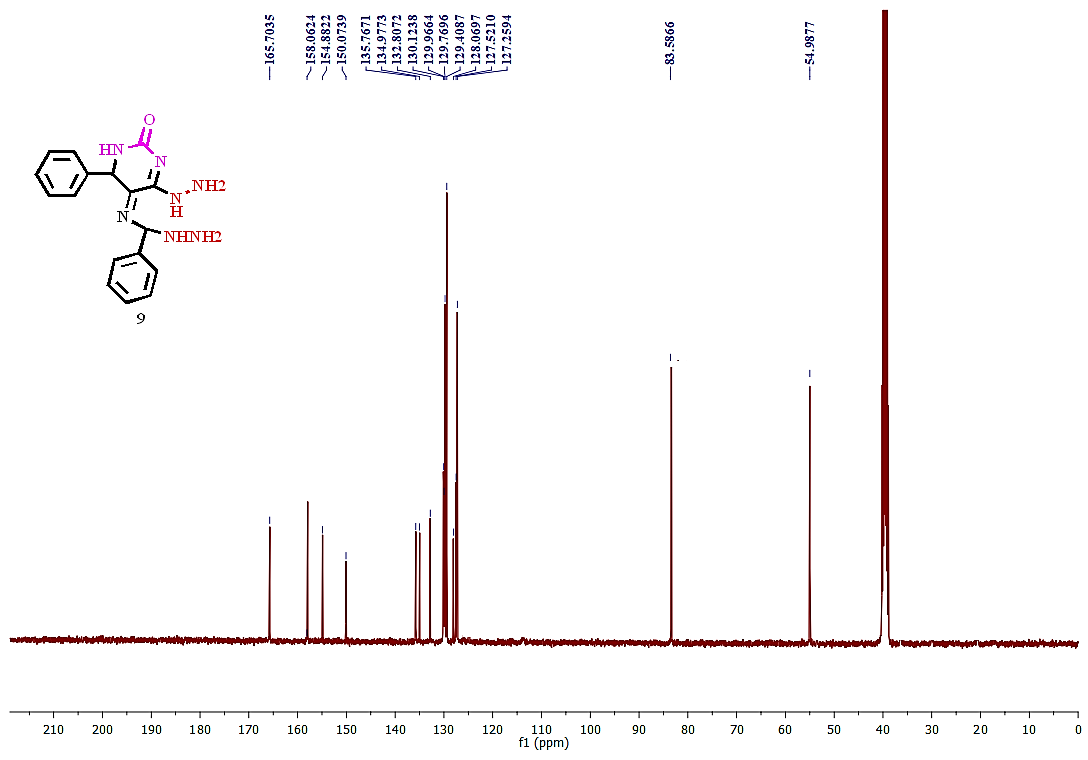
**

**Fig. 24**: **^13^C-NMR Spectrum of compound 9**

**
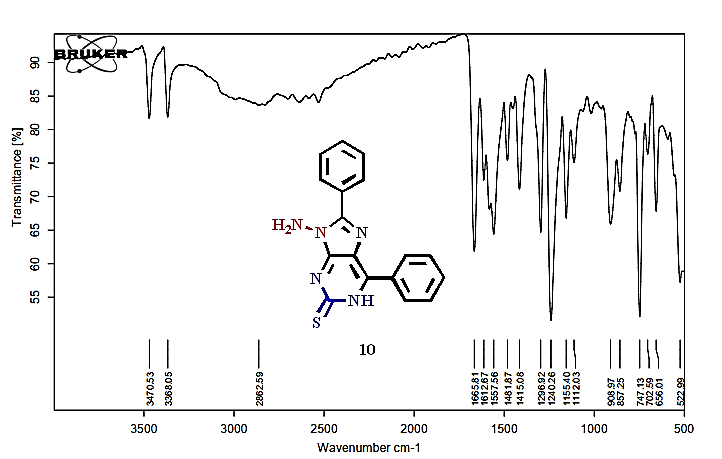
**

**Fig. 25**: **IR Spectrum of compound 10**

**
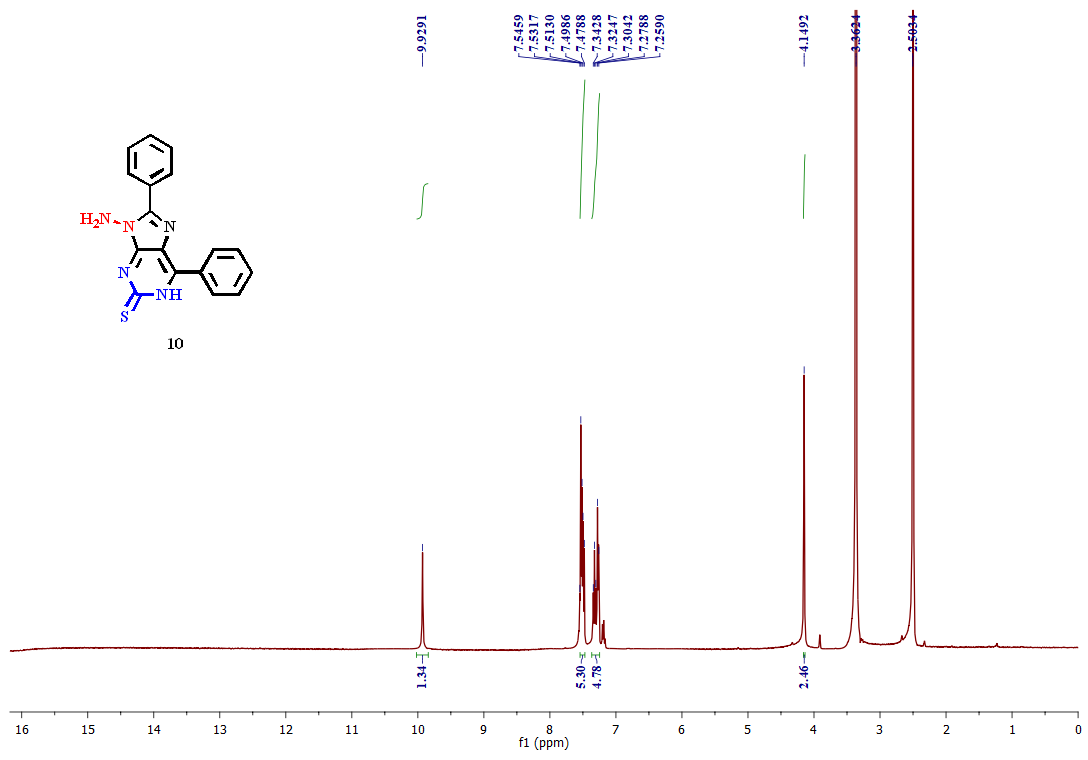
**

**Fig. 26**: **^1^H-NMR Spectrum of compound 10**

**
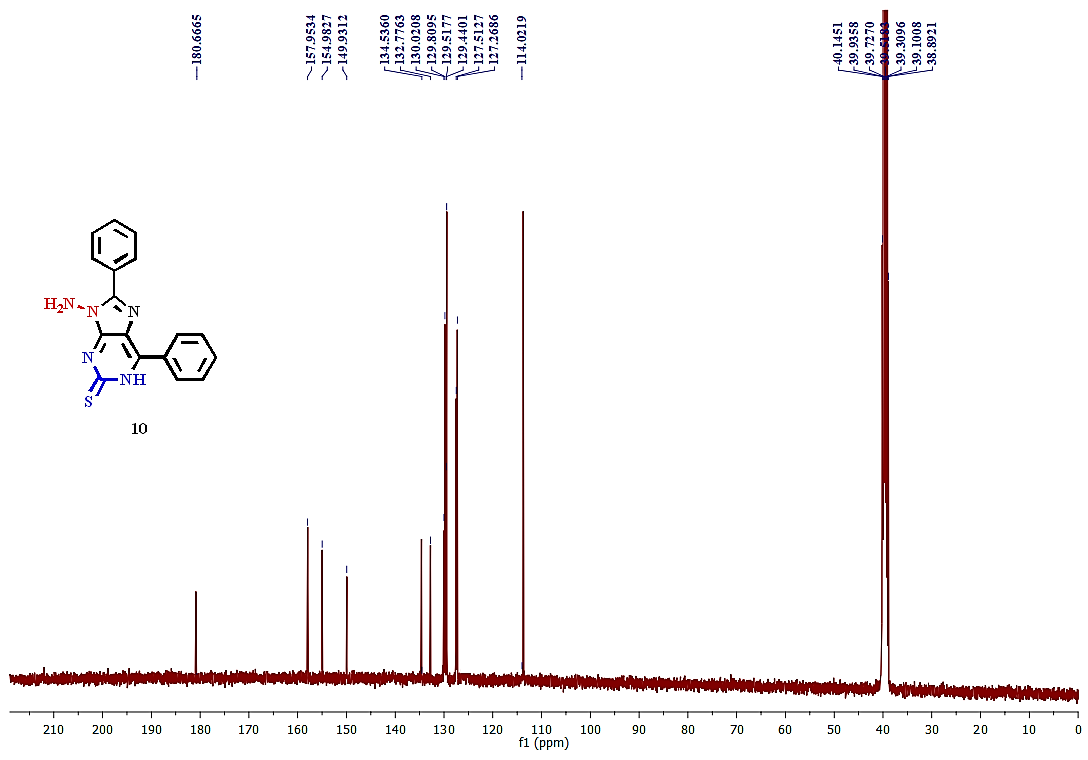
**

**Fig. 27**: **^13^C-NMR Spectrum of compound 10**

**
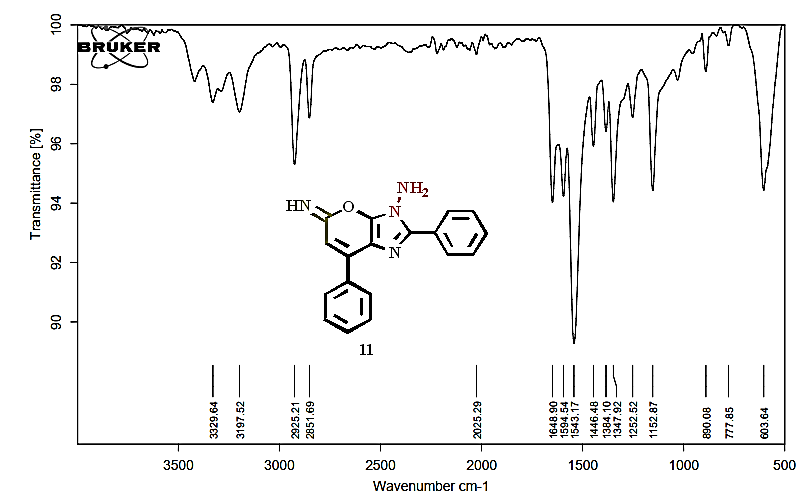
**

**Fig. 28**: **IR Spectrum of compound 11**

**
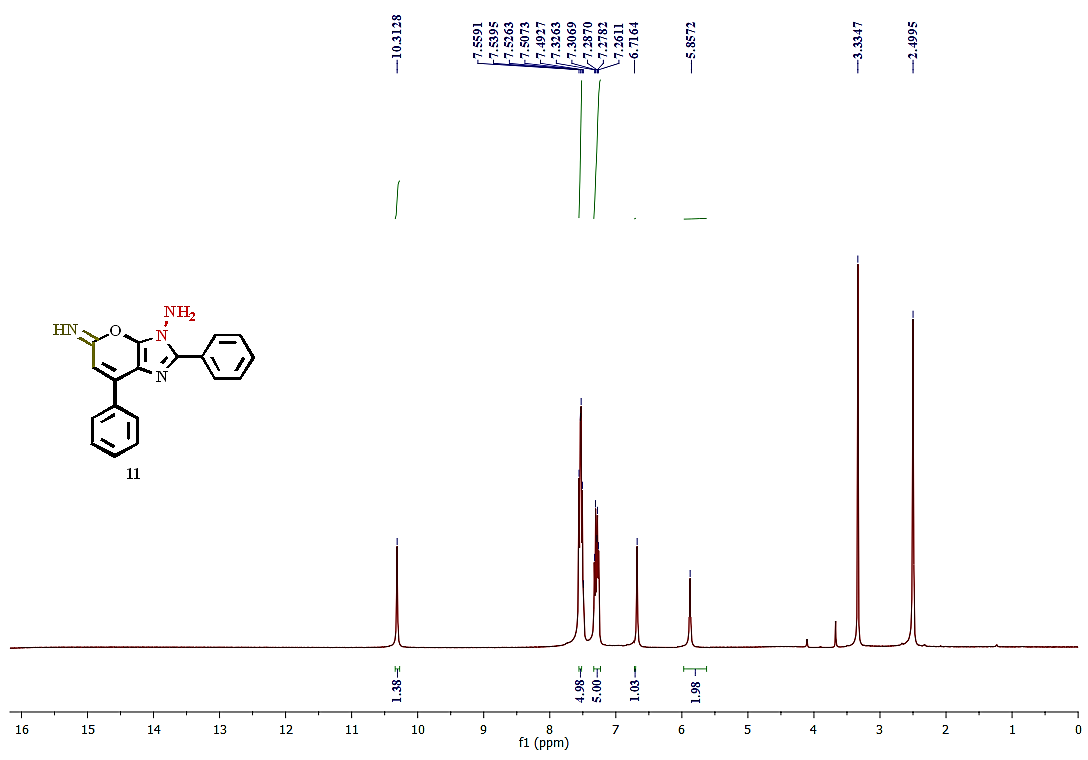
**

**Fig. 29**: **^1^H-NMR Spectrum of compound 11**

**
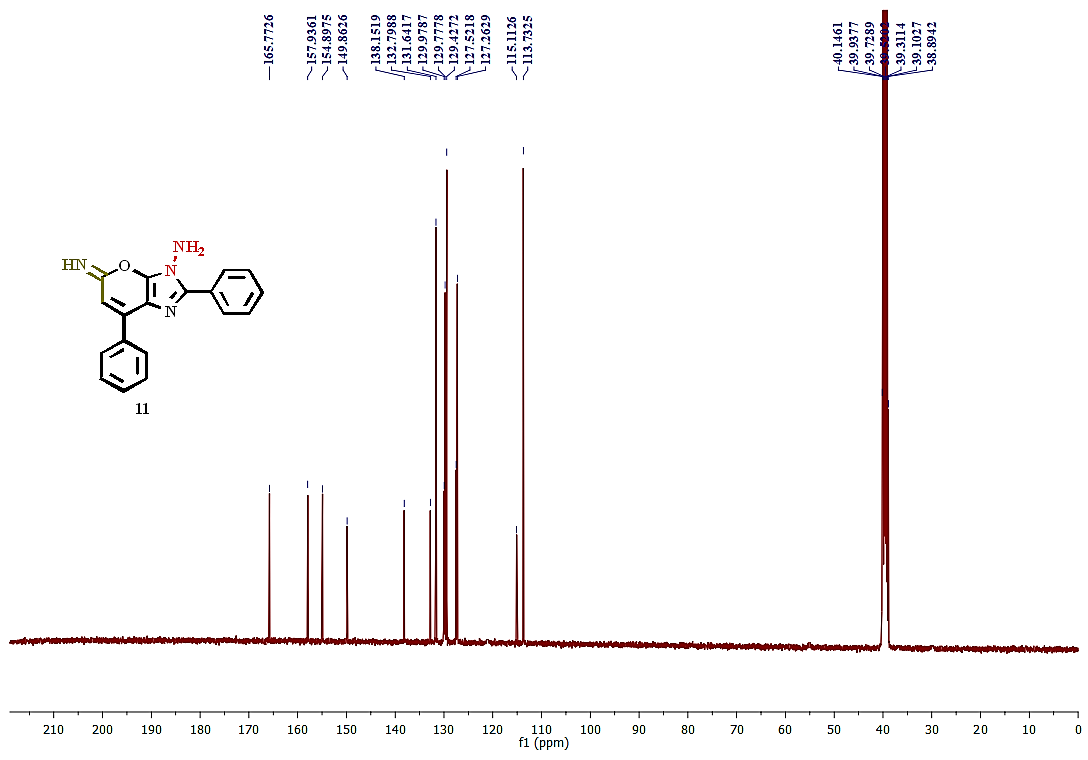
**

**Fig. 30**: **^13^C-NMR Spectrum of compound 11**

**Table 1S:** DFT calculations are used to determine the equilibrium geometric properties of the compound, including bond lengths (Å), bond angles (˚), dihedral angles (˚), total energy (k cal/mol), and dipole moment (3).

| Bond length (Å) | | | | | | | | | | | | | | | | |
| --- | --- | --- | --- | --- | --- | --- | --- | --- | --- | --- | --- | --- | --- | --- | --- | --- |
| N1-C2  C2-S7  C2-N6  C5-N6  C5-O9  C4-C5  C3-C4  N1-C3 | 1.335  1.692  1.341  1.372  1.208  1.363  1.349  1.344 | | C11-O15  C11-O12  C12-C13  C13-C14  C4-C14  C14-C16  C16-C17  C17-C18 | | | 1.212  1.352  1.331  1.492  1.516  1.515  1.346  1.342 | C19-C20  C20-C21  C16-C21  C12-O23  C24-O23  C24-N22  C13-N22  C24-C25 | | | 1.341  1.342  1.346  1.349  1.366  1.358  1.353  1.345 | | | C26-C27  C27-C28  C28-C29  C29-C30  C25-C30  C25-C26  C18-C19  C3-C11 | | 1.343  1.342  1.341  1.342  1.347  1.347  1.341  1.365 | |
| Bond angle (˚) | | | | | | | | | | | | | | | | |
| N1C2S7  N6C2S7  N1C2N6  C2N6C5  N6C5O9  N6C5C4  C3C4C5  N1C3C4  C2N1C3  C11C3N1  C25C30C29 | | 120.82  120.85  118.33  120.85  119.17  120.02  118.07  120.69  122.04  117.07  121.09 | | | C5C4C14  C11C3C4  C3C4C14  C4C14C13  C14C13C12  C13C12C11  C3C11C12  C3C11O15  C12C11O15  C11C12O23  C28C29C30 | | | | 118.70  122.23  123.22  108.15  124.29  123.02  118.84  122.04  119.11  126.54  120.09 | | | C12O23C24  O23C24N22  C24N22C13  N22C13C12  N22C13C14  N22C24C25  O23C24C25  C24C25C26  C25C26C27  C26C27C28  C7C28C29  C24C25C30 | | 103.47  112.39  103.79  109.91  125.79  122.00  125.60  122.01  120.98  120.18  119.48  119.82 | | |
| Dihedral angles (˚) | | | | | | | | | | | | | | | | |
| S7C2N1C3  S7C2N6C5  O9C5N6C2  O9C5C4C3  O9C5C4C14  C2N1C3C11  C2N1C3C4  N1C3C11C12  N1C3C4C14  O15C11C3N1  O15C11C3C4 | | -179.73  179.46  -179.55  179.68  -0.788  178.77  -0.079  -176.88  -179.28  2.01  -179.15 | | O15C11C12O23  C13C14C4C5  C3C11C12O23  C11C12O23C24  C24N22C13C14  N22C13C14C16  C13C14C16C17  C13C14C16C21  C14C16C21C20  C14C16C17C18 | | | | -2.625  175.51  176.30  -178.51  -179.62  63.15  -116.92  62.79  -179.99  -179.96 | | | C13N22C24C25  N22C24C25C26  O23C24C25C26  O23C24C25C30  C30C25C24N22  C24C25C30C29  C24C25C26C27  C5C4C14C16  C12O23C24C25  O15C11C12C13 | | | | | -179.23  -0.205  -179.67  0.150  179.62  -179.79  179.78  -64.67  179.32  -179.07 |
| Total energy/ k cal/mol  Total dipole moment/D | | | | | | | -147855.296  3.028 | | | | | | | | | |

**Table 2S:** DFT calculations are used to determine the equilibrium geometric properties of the compound, including bond lengths (Å), bond angles (˚), dihedral angles (˚), total energy (k cal/mol), and dipole moment (4).

| Bond length (Å) | | | | | | | | | | | | | | | | |
| --- | --- | --- | --- | --- | --- | --- | --- | --- | --- | --- | --- | --- | --- | --- | --- | --- |
| C1-C5  C5-N6  N6-N9  N9-C8  C7-C8  C5-C7  N9-C10  C10-C11  C11-C12 | 1.498  1.352  1.362  1.346  1.346  1.346  1.345  1.343  1.342 | | C12-C13  C13-C14  C14-C15  C15-C10  C14-C15  C7-O21  O21-C22  C22-C23  C23-C24 | | | 1.340  1.342  1.341  1.343  1.523  1.359  1.357  1.337  1.506 | C24-C25  C25-C26  C26-C27  C27-C28  C28-C29  C29-C30  C25-C30  C22-O38  C37-O38 | | | 1.513  1.345  1.342  1.341  1.342  1.341  1.346  1.349  1.366 | | | C37-N36  N36-C23  C37-C39  C39-C40  C40-C41  C41-C42  C42-C43  C43-C44  C40-C39 | | 1.357  1.353  1.346  1.347  1.342  1.341  1.340  1.343  1.347 | |
| Bond angle (˚) | | | | | | | | | | | | | | | | |
| C1C5N6  C1C5C7  C5C7O21  C11C10N9  C15C10N9  C10N9N6 | | 123.85  127.63  127.73  119.88  119.87  126.52 | | | C23C24C25  C24C25C26  C24C25C30  N9C8C24  C24C23N36  O21C22O38 | | | | 110.01  121.11  120.47  128.79  125.97  125.19 | | | C37C39C40  C37C39C44  N36C37C39  O38C37C39  C10N9C8  C8C24C25 | | 119.72  122.14  121.82  125.49  122.12  111.99 | | |
| Dihedral angles (˚) | | | | | | | | | | | | | | | | |
| C1C5C7O21  C1C5C7C8  C1C5N6N9  C5N6N9C10  C10N9C8C7  C10N9C8C24  C11C10N9N6  C11C10N9C8 | | 0.315  178.51  -179.74  167.95  -168.57  11.04  -65.16  99.16 | | C30C25C24C8  C30C25C24C23  C26C25C24C8  C26C25C24C23  C25C24C23N36  C39C37O38C22  C39C37N36C23  C15C10N9C8 | | | | -55.54  63.07  124.71  -116.67  53.14  -177.73  178.01  -78.37 | | | C44C39C37N36  C44C39C37O38  C40C39C37O38  C40C39C37N36  C15C10N9N6  N9C8C24C25  N9C8C24C23 | | | | | 0.543  179.23  -0.431  -179.12  117.32  -55.47  -175.97 |
| Total energy/ k cal/mol  Total dipole moment/D | | | | | | | -155327.314  1.904 | | | | | | | | | |

**Table 3S**: DFT calculations are used to determine the equilibrium geometric properties of the compound, including bond lengths (Å), bond angles (˚), dihedral angles (˚), total energy (k cal/mol), and dipole moment (9).

| Bond length (Å) | | | | | | | | | | | | | | | | |
| --- | --- | --- | --- | --- | --- | --- | --- | --- | --- | --- | --- | --- | --- | --- | --- | --- |
| C1-C2  C2-C3  C3-C4  C4-C5  C5-C6  C1-C6  C3-C12  C12-N13  C14-N15 | 1.342  1.346  1.345  1.342  1.341  1.340  1.515  1.441  1.404 | | C14-O18  N15-C16  C16-C17  C12-C17  C17-N19  N19-C34  C16-N21  N21-C22 | | | 1.206  1.262  1.349  1.518  1.352  1.445  1.346  1.347 | C25-C26  C26-C27  C27-C28  C28-C22  C34-C35  C35-C36  C36-C37  C37-C38 | | | 1.342  1.341  1.343  1.345  1.516  1.346  1.342  1.341 | | | C35-C40  C34-N46  N46-N47  C38-C39  C39-C40  C22-C24  C24-C25  N13-C14 | | 1.345  1.445  1.389  1.340  1.342  1.345  1.342  1.373 | |
| Bond angle (˚) | | | | | | | | | | | | | | | | |
| C2C4C46  C5C4C46  N8C9O12  C10C9O12  C10C14C15  C10C14C28  C15C14C28  C14C15C20  C14C15C16 | | 120.87  120.88  118.35  121.65  115.47  116.91  109.57  119.48  123.31 | | | C15C20O21  N19C20O21  N19C18O23  N17C18O23  C18N17C24  C16N17C24  N17C24C42  C15C16N25  N17C16N25 | | | | 121.41  118.61  118.91  124.06  119.18  121.72  110.82  117.34  118.15 | | | C14C28C14  C14C28C37  C28C37O38  N36C37O38  N36C34O39  N30C34O39  C29N30C35  C54C35N30 | | 120.86  122.15  122.33  117.68  119.06  124.06  122.03  110.91 | | |
| Dihedral angles (˚) | | | | | | | | | | | | | | | | |
| C16N21C22C24  C16N21C22C28  N15C16N21C22  C17C16N21C22  O18C14N15C6  O18C14N13C12  N19C17C16N21  C3C12C17N19  C3C12C17C16 | | 134.69  -48.24  20.48  -159.29  177.99  177.10  10.83  -75.04  111.87 | | C4C3C12C17  C4C3C12N13  C12C3C2C1  C12C3C4C5  C12C17N19C34  C16C17N19C34  C17N19C34C35  C17N19C34N46  N19C34C35C40 | | | | -40.17  83.29  179.88  179.99  -0.595  172.85  79.65  -158.97  38.69 | | | N46C34C35C36  N47N46C34C35  N47N46C34N19  N19C34N46N47  N46C34C35C40  C2C3C12C17  C2C3C12N13  N19C34C35C36  C3C12N13C14 | | | | | 99.65  -55.75  -177.31  -177.31  -79.81  141.28  -95.27  -141.85  -115.33 |
| Total energy/ k cal/mol  Total dipole moment/D | | | | | | | -153791.243  7.966 | | | | | | | | | |

**Table 4S:** DFT calculations are used to determine the equilibrium geometric properties of the compound, including bond lengths (Å), bond angles (˚), dihedral angles (˚), total energy (k cal/mol), and dipole moment (10).

**Table 5S:** equilibrium geometric parameters such as bond lengths (Å), bond angles (˚), dihedral angles (˚), total energy (kcal/mol), and the dipole moment of compound (11) were obtained using DFT calculations.

| Bond length (Å) | | | | | | | | | | | | | | | | |
| --- | --- | --- | --- | --- | --- | --- | --- | --- | --- | --- | --- | --- | --- | --- | --- | --- |
| C1-O2  O2-C3  C3-C4  C4-C5  C5-C6  C1-C6  C1-N7 | 1.355  1.349  1.337  1.354  1.359  1.347  1.288 | | C3-N8  N8-C9  C9-N10  C4-N10  N8-N35  C9-C18  C18-C19 | | | 1.342  1.349  1.351  1.346  1.357  1.351  1.351 | C19-C20  C20-C21  C21-C22  C22-C23  C18-C23  C5-C12 | | | 1.343  1.339  1.338  1.343  1.348  1.365 | | | C12-C13  C13-C14  C14-C15  C15-C16  C16-C17  C12-C17 | | 1.355  1.343  1.337  1.338  1.343  1.353 | |
| Bond angle (˚) | | | | | | | | | | | | | | | | |
| N7C1O2  N7C1C6  O2C3N8  C3N8N35  C9N8N35 | | 117.43  120.87  124.73  124.62  130.79 | | | N8C9C18  C18C9N10  C9C18C23  C9C18C19  C5C4N10 | | | | 128.83  120.19  124.03  119.74  129.93 | | | C4C5C12  C6C5C12  C5C12C13  C5C12C17 | | 124.95  122.09  123.07  123.65 | | |
| Dihedral angles (˚) | | | | | | | | | | | | | | | | |
| C3O2C1N7  C5C6C1N7  C4C3N8N35  O2C3N8N35  N10C9N8N35  C18C9N8N35 | | 179.53  -178.39  -169.38  9.236  168.18  -13.21 | | N8C9C18C23  N8C9C18C19  N10C9C18C19  N10C9C18C23  C3N8C9C18  C6C5C12C13 | | | | -1.53  179.14  -2.38  176.96  176.34  -178.59 | | | C18C9N10C4  C12C5C4N10  C12C5C4C3  C4C5C12C17  C6C5C12C17 | | | | | -176.85  -0.46  179.51  -1.89  -1.99 |
| Total energy/ k cal/mol  Total dipole moment/D | | | | | | | -116566.493  3.190 | | | | | | | | | |
